# Correlative 3D imaging method for analysing lesion architecture in susceptible mice infected with *Mycobacterium tuberculosis*

**DOI:** 10.1242/dmm.052185

**Published:** 2025-03-26

**Authors:** Caroline G. G. Beltran, Jurgen Kriel, Stefan M. Botha, Margaret B. Nolan, Alessandro Ciccarelli, Ben Loos, Maximiliano G. Gutierrez, Gerhard Walzl

**Affiliations:** ^1^ Department of Biomedical Sciences, Faculty of Medicine and Health Sciences, Stellenbosch University, Cape Town 7505, South Africa; ^2^ South African Medical Research Council Centre for Tuberculosis Research, Division of Molecular Biology and Human Genetics, Faculty of Medicine and Health Sciences, Stellenbosch University, Cape Town 7501, South Africa; ^3^ Central Analytical Facilities, Faculty of Medicine and Health Sciences, Stellenbosch University, Cape Town 7505, South Africa; ^4^ The Francis Crick Institute, London NW1 1AT, UK; ^5^ Department of Physiological Sciences, Stellenbosch University, Stellenbosch 7602, South Africa

**Keywords:** CLARITY, Three-dimensional, Light-sheet fluorescence microscopy, Serial block face electron microscopy, Tuberculosis, Granuloma, Virulence

## Abstract

Tuberculosis (TB) is characterized by the formation of heterogeneous, immune-rich granulomas in the lungs. Host and pathogen factors contribute to this heterogeneity, but the molecular and cellular drivers of granuloma diversity remain inadequately understood owing to limitations in experimental techniques. In this study, we developed an approach that combines passive CLARITY (PACT)-based clearing with light-sheet fluorescence microscopy to visualize lesion architecture and lung involvement in *Mycobacterium tuberculosis*-infected C3HeB/FeJ mice. Three-dimensional rendering of post-mortem lungs revealed critical architectural features in lesion development that traditional thin-section imaging could not detect. Wild-type *M. tuberculosis* infection resulted in organized granulomas, with median sizes increasing to 3.74×10^8^ µm^3^ and occupying ∼10% of the total lung volume by day 70 post-infection. In contrast, infection with the avirulent ESX-1 deletion mutant strain resulted in diffuse and sparsely organized CD11b recruitment (median size of 8.22×10^7^ µm^3^), primarily located in the lung periphery and minimally involving the airways (0.23% of the total lung space). Additionally, we present a method for volumetric correlative light and electron microscopy, enabling tracking of individual immune cell populations within granulomas.

## Teaser

**Summary:** A novel 3D imaging approach combining passive CLARITY clearing and volumetric electron microscopy provides insight into how tuberculosis granulomas form, evolve and spatially organize, revealing dynamic host–pathogen interactions in a susceptible mouse model.

## INTRODUCTION

Granulomas are complex, multicellular and pathological immune responses localized in the lungs of patients with tuberculosis (TB), representing the hallmark of this disease. It is well established that infection with the bacterial pathogen *Mycobacterium tuberculosis* (*Mtb*) results in a wide range of pathological heterogeneity (Lenaerts et al., 2015; Ravimohan et al., 2018), even within the same patient, and that lesion-to-lesion variation determines different outcomes of infection control (Lanoix et al., 2015). The host–pathogen interface plays a dominant role in TB and determines the severity of lung damage (Ehlers and Schaible, 2012). Patients can present with cavitation, fibrosis or nodular infiltrates, often with a mixture of these pathologies (Ravimohan et al., 2018). It remains challenging to comprehensively characterize this variability and fully understand the implications on the ability of the host to contain the infection and return to normal tissue structure and function. Assessing new drugs, host-directed therapies and vaccines is still reliant on animal models, most notably murine models, yet suffers from limited contextual information, specifically, regarding molecular and cellular drivers of lesion heterogeneity and the degree and severity of lung involvement. Most studies utilize 2D thin sectioning and/or whole-organ homogenized tissue assessment, providing limited spatial and contextual information. Although classic light and fluorescent microscopy can be used to observe local host–pathogen interactions in the infected organs, these methods limit the analysis to small areas. Consequently, histologic specimens are subject to fragmented information and sampling bias, with the overall 3D anatomical context often not considered. Three-dimensional spatial analysis of infected tissues could allow quantitative profiling while maintaining tissue architecture and identify key biological patterns that facilitates the identification and profiling of important clinical features during TB. Advancements in 3D imaging techniques have already significantly deepened our understanding of TB granulomas, revealing structural complexities that were previously underappreciated in 2D studies (Ordonez et al., 2021). Notably, studies utilizing micro-computed tomography (microCT) to reconstruct the 3D architecture of necrotic TB granulomas in human lungs challenged the traditional perception of granulomas as simple spherical structures (Wells et al., 2021, 2022). Studies utilizing zebrafish have also been pivotal in providing early insights into the complex interplay between the host and pathogen during granuloma formation in 3D (Davis et al., 2002; Swaim et al., 2006). The optical transparency in the larval stages led to its early adoption as a model for the study of disease, allowing real-time imaging of macrophage aggregation even in the absence of lymphocyte recruitment, and the mechanisms by which bacteria disseminate within granulomas, contributing to our understanding of the pathogenesis of tuberculosis (Davis et al., 2002). Although zebrafish offer several key advantages for studying granuloma formation, their lack of lungs limits their full applicability for modelling certain aspects of TB. Nonetheless, these studies highlight the power of visualizing disease processes in transparent systems, providing valuable insights into the underlying mechanisms and informing the development of new therapeutic strategies.

The surge in tissue clarification techniques has unveiled novel ways of interrogating tissue architecture in various biological tissues in 3D space (Du et al., 2018; Richardson and Lichtman, 2015; Woo et al., 2016; Yu and Zhu, 2018). These techniques are particularly attractive to study diseases for which spatial heterogeneity is typical of the disease. Developments in tissue clarification and deep-tissue imaging are paving the way for more appropriate ways to visualize tissue without the loss of contextual information (Mzinza et al., 2018; Rocha et al., 2019; Yu et al., 2018; Zhu et al., 2013). Clearing techniques have been used extensively to study the intact structure of the brain, enabling unprecedented visualization of intricate neural architecture (Epp et al., 2015; Liu et al., 2016; Yu and Zhu, 2018); however, their application in the study of infectious diseases, particularly TB, remains comparatively underutilized to a handful of studies (Cronan et al., 2015; Francis et al., 2020; Kang et al., 2020). These studies have demonstrated the benefit of retaining the 3D spatial context for modelling TB pathophysiology. In particular, a study describing a procedure that combined mesoscopy with CUBIC Acid-Fast staining achieved high-resolution 3D imaging of infected lungs and proved highly effective in enabling the study of *Mtb* infection with different strains (Francis et al., 2020). Although this study achieved sub-cellular resolution over large fields of view, certain limitations regarding the specificity of Rhodamine O and Auramine staining for *Mtb* sub-populations, as well as slow scan speeds of confocal laser scanning microscopy, highlight the need for complementary methods to overcome some of these challenges.

The integration of aqueous-based clearing techniques such as passive CLARITY (PACT) (Yang et al., 2014), with the advantages of light-sheet fluorescent microscopy (LSFM) imaging, owing to its faster, less damaging acquisition and greater imaging depth, could provide a powerful platform for TB pathophysiology assessment. PACT offers the advantage of preserving endogenous fluorescence while allowing for flexible immunofluorescent labelling. When applied to a highly relevant TB infection model, such as the C3HeB/FeJ mouse strain, this approach enhances the ability to draw meaningful conclusions about disease mechanisms. This mouse model is particularly valuable as it recapitulates the heterogeneity of human TB pathology, including the manifestation of three distinct lesion types during low-dose infection (Driver et al., 2012; Harper et al., 2012; Irwin et al., 2015; Lanoix et al., 2015; Pan et al., 2005). Unlike other mouse strains, which often develop diffuse or poorly organized granulomas, C3HeB/FeJ mice exhibit structured lesions with a central necrotic core (Driver et al., 2012; Harper et al., 2012). This characteristic makes the model ideal for studying the relationship between granuloma architecture, bacterial growth dynamics and disease progression. Additionally, the rapid bacterial replication observed in these mice allows researchers to explore how granuloma integrity influences bacterial containment and how granuloma breakdown facilitates disease dissemination. This model provides critical insights into TB pathogenesis, particularly in the context of severe, progressive disease, offering a more accurate representation of human TB than other commonly used mouse strains.

Here, we developed a PACT-based clearing and LSFM approach to investigate and map the formation of lesions in 3D in TB-infected C3HeB/FeJ mouse lungs over time. We investigated the dynamics of an ESX-1 deletion *Mtb* mutant and its influence during early and late lesion formation. Following identification of regions of interest (ROIs) in 3D, lesions were excised and prepared for serial block face electron microscopy (SBF-EM) to provide high ultrastructural content from clarified tissue, termed volumetric correlative light electron microscopy (vCLEM).

## RESULTS

### Bacterial burden and lung pathology are reduced in C3HeB/FeJ mice following aerosol infection with *Mtb* H37Rv ΔRD1

**Fig. 1.**
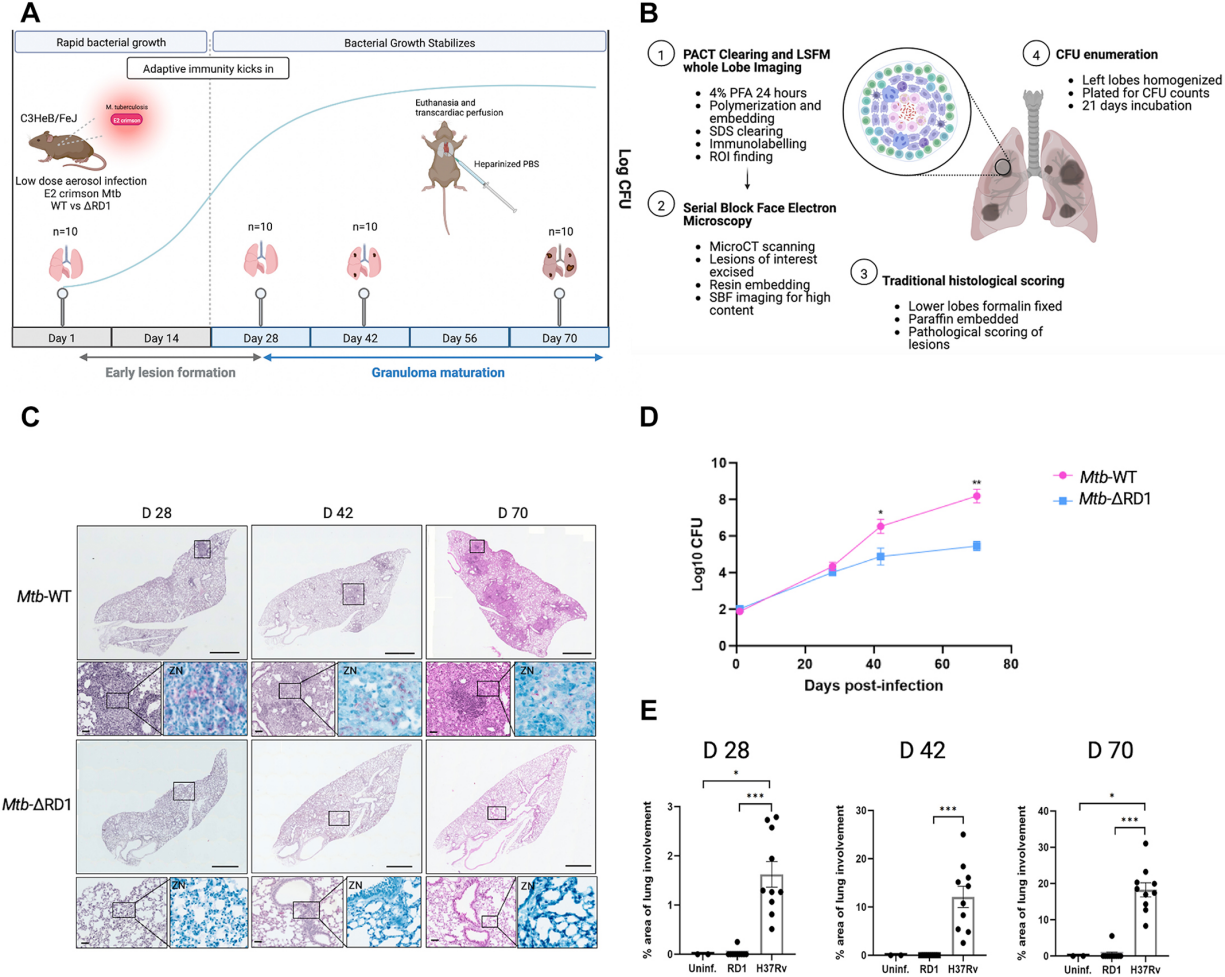
**Multi-modal imaging and quantification of tuberculosis infection in C3HeB/FeJ mouse lungs.** (A) Whole lung lobe imaging workflow. A C3HeB/FeJ murine low-dose tuberculosis (TB) infection model was used. Forty/group of 8- to 10-week-old C3HeB/FeJ mice were infected via inhalation exposure system with <100 colony forming units (CFU) of either E2Crimson *M. tuberculosis* (*Mtb*) wild type (WT) (*Mtb*-WT) or E2Crimson *Mtb* ESX-1 deletion mutant (*Mtb-*ΔRD1) – *Mtb* transformed with a PTEC-19 plasmid containing an E2Crimson reporter. Ten mice/group were euthanized 1 day post-infection to confirm delivery dose. Infection was allowed to progress over time. Lungs were extracted at day 28, 42 and 70 post-infection (ten mice/group) following transcardiac perfusion with heparinized phosphate buffered saline (PBS) to remove red blood cells. (B) Overview of lung lobe allocation and procedure for each assay. (1) Right cranial and middle lobes were perfused post-extraction with 4% paraformaldehyde and fixed for 24 h at room temperature. Right cranial lobes were processed for passive CLARITY (PACT)-based clearing and immunolabelling before imaging with light-sheet fluorescent microscopy (LSFM). (2) Regions of interest (ROIs) imaged at higher magnification using LSFM were scanned using microCT, excised and resin embedded prior to serial block face electron microscopy (SBF). (3) Right caudal lobes were placed in 10% formalin and fixed for 24 h prior to paraffin embedding and sectioning and Haematoxylin and Eosin (H&E) staining for standard histology. (4) Left lobes were removed following cardiac perfusion, placed in 4 ml PBS, homogenized and plated for CFU determination. (C) Representative histological (H&E) staining images of lower-right lobes from infected mice at day (D)28, D42 and D70. Scale bars: 1000 μm (upper panels) and 50 μm (lower, zoomed-in panels). ‘ZN’ panels correspond to parallel histological sections of the same region stained using Ziehl–Neelsen staining. (D) Bacterial growth and lung pathology in the lungs of C3HeB/FeJ mice exposed to a low-dose aerosol infection of *Mtb* H37Rv WT versus ΔRD1 mutant over time (D28, D42 and D70 post-infection). Data points represent mean log_10_ CFU±s.e.m. of ten animals per time point. (E) Percentage lung involvement at D28, D42 and D70 for uninfected animals (uninf.), ΔRD1 mutant (RD1) and H37Rv WT, indicating cell infiltration. Data are from two animals (uninf.) and ten animals per strain (RD1 and WT) at each time point. Data points represent s.e.m. Significance testing was done using a Kruskal–Wallis test with post-hoc Dunn's test and Bonferroni correction (*P<0.05; **P<0.01; ***P<0.001).

We used a murine model of TB that more closely represents human pathology by employing the C3HeB/FeJ mouse model and a low-dose aerosol infection model using E2Crimson fluorescently labelled *Mtb* H37Rv wild type (E2Crimson *Mtb* WT) and E2Crimson fluorescently labelled *Mtb* H37Rv ESX-1 deletion mutant (E2Crimson *Mtb* ΔRD1) (Fig. 1A). Forty mice per group were infected with 100 colony-forming units (CFU), and the infection was allowed to progress over time. At defined time points (1, 2, 42 and 70 days post-infection), ten mice per group were euthanized, and red blood cells were removed via transcardiac perfusion, after which lung lobes were processed for PACT and immunolabelling, and imaged using LSFM (Fig. 1B). PACT-based clearing allowed gentle and slow clearing, thereby preserving sufficient lipid content to enable contrast with electron microscopy. We therefore identified regions/lesions of interest and further processed these for SBF-EM. Histological analysis and CFU determination (Fig. 1C) were conducted on the lower-right caudal lobes and left lung lobes, respectively.

Bacterial growth kinetics of the E2Crimson *Mtb* WT and E2Crimson *Mtb* ΔRD1 were monitored in the lungs over time. CFU counts showed consistent growth for both the E2Crimson *Mtb* WT and E2Crimson *Mtb* ΔRD1 mutant early in the infection (day 28). However, there was significantly reduced growth observed in the mutant at day 42 and day 70 post-infection (Fig. 1D). Lung histopathology analysis (Fig. 1B) showed early evidence of lesions at day 28 in E2Crimson *Mtb* WT-infected mice, further expanding across the lungs at later time points. By contrast, E2Crimson *Mtb* ΔRD1-infected mice showed little evidence of cell infiltration, even at later time points. Quantification of the total lung inflammation using traditional pathological scoring showed significant differences across all time points between the E2Crimson *Mtb* WT and the E2Crimson *Mtb* ΔRD1 mutant (Fig. 1E). The E2Crimson *Mtb* ΔRD1 mutant had significantly less lung involvement, even at the later time points. Of the ten mice analysed, only a small subset showed any lung involvement (Table S1), despite consistent growth of the strain as indicated by CFU analysis. The E2Crimson *Mtb* WT-infected mice showed a median of 1.5% lung involvement at day 28, increasing to 12% by day 42 and 18% by day 70. E2Crimson *Mtb* ΔRD1-infected mice showed almost no lung involvement throughout the infection period, as assessed by Haematoxylin and Eosin (H&E) staining (Fig. 1E).

### LSFM allows the visualization of lesion development in whole lungs during *Mtb* WT infection

Following successful clearing of infected lung lobes using PACT and refractive index matching solution (RIMS) matching with HistoDenz™, we confirmed that the E2Crimson reporter from *Mtb* was detectable in unlabelled tissue and determined the level of resolution obtained using LSFM. A cleared lung lobe infected with E2Crimson *Mtb* WT (42 days post-infection) was imaged using a LaVision Ultramicroscope II Light Sheet Microscope (Miltenyi Biotec) using low magnification (0.63× zoom) and static light sheet (12 µm thickness) to generate an overview of the entire lobe, and further imaged at a higher resolution using 6.3× zoom and dynamic horizontal focusing.

**Fig. 2.**
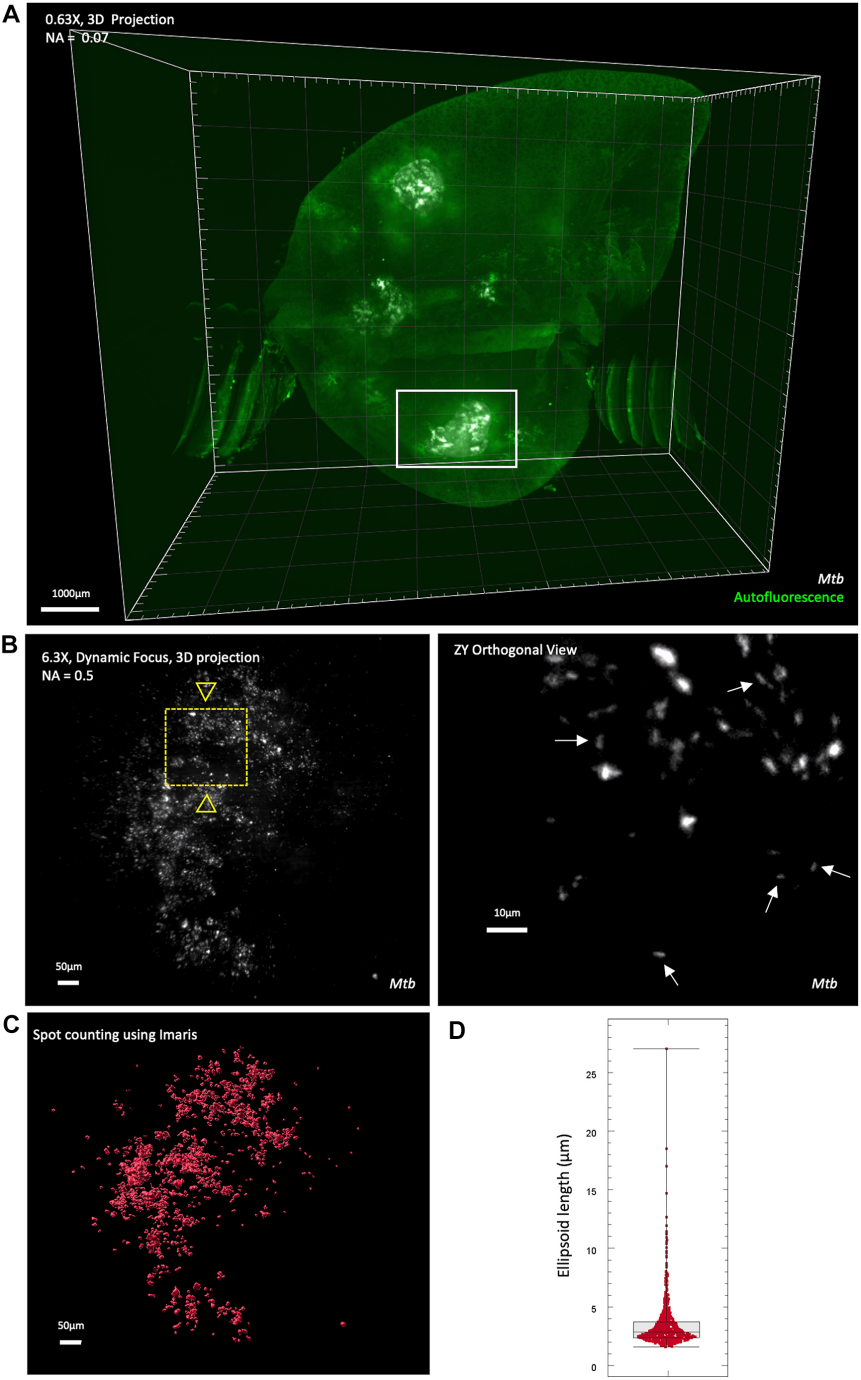
**3D view of an infected (D42) lung lobe using LSFM.** (A) Whole-lobe imaging using 0.63× zoom, showing the merged signal from the *Mtb* E2Crimson reporter (white) and tissue autofluorescence (green). Image was acquired with 100% sheet width (12 µm thickness), 10 µm *z*-step size and 100 ms exposure time, and illumination from left and right side. Scale bar: 1000 µm; numerical aperture (NA)=0.07. (B) Left: 3D view of an ROI (white box, panel A) imaged at higher resolution (dynamic focus with 6.3× zoom and NA of 0.5) to identify the *Mtb* E2Crimson reporter in higher detail. Image was acquired with 100% sheet width (7 µm thickness) at 2 µm *z*-step interval, 100 ms exposure, and illumination from left and right side. Scale bar: 50 µm. Right: the ZY orthogonal view (position 183 µm) is referring to a subregion of the volume shown on the left (indicated by the yellow dashed line box), showing individual *Mtb* cells (white arrows). ZY projections are enlarged roughly fivefold relative and image gamma modified to enhance display of dim features. Scale bar: 10 µm. (C) Individual cell counting using Imaris spot counting feature to identify and count individual cells from the 6.3× stack. (D) Box and whisker plot showing the average length (ellipsoid axis length in µm) of the *Mtb* cells identified during spot counting. Box plot represents the interquartile range, with whiskers as the maximum and minimum values.

We first established background autofluorescence levels, indicating a high level of tissue autofluorescence in the green channel (illumination with 488 nm laser line) (Fig. 2A), which could be used as a marker for general airway and lung architecture without the requirement for immunolabelling. The E2Crimson reporter was visible in both the red and far-red (illumination with the 594 and 647 nm laser lines, respectively). The near infra-red channel (790 nm) was identified as having no autofluorescence, from either the tissue or the E2Crimson reporter, and thus selected for further immunostaining using near infra-red fluorophores (Fig. S2). The images showed appropriate imaging depth and enabled visualization of the entire lung anatomy (Fig. 2A; Movie 1), as well as infected zones containing *Mtb* (Fig. 2B). We further imaged a section of infected space using a higher magnification (6.3×) and dynamic focus, allowing deeper visualization of infecting bacteria (Fig. 2B). Optical sectioning allowed interrogation of different depths of the lung tissue, and individual *Mtb* cells were visible in the orthogonal stack (white arrows, Fig. 2B; orthogonal slices). However, the *Mtb* signal was predominantly visible as a mass, in which individual spots could not be resolved accurately. Imaris cell counting was utilized to determine the total number of cells in a region (Fig. 2C), and the average spot length was calculated using ellipsoid axis length. On average, spot counting identified the mean length of detected spots as 4 µm (Fig. 2D), with several cell spots longer than 10 µm, likely representing cell aggregates rather than single cells. These data indicate the limitation to identifying and counting individual *Mtb* cells with this imaging approach.

**Fig. 3.**
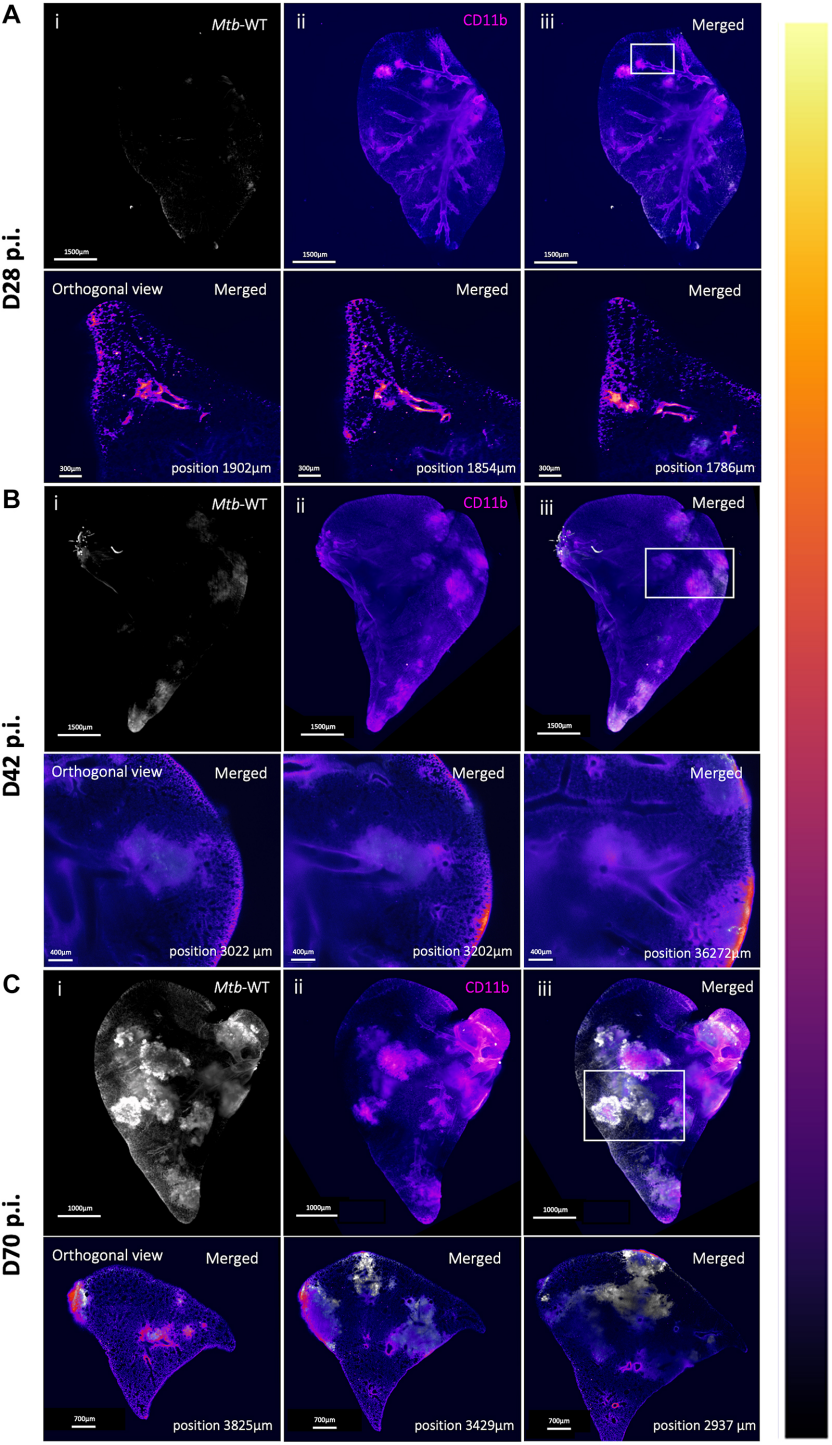
**LSFM imaging of PACT-based cleared C3HeB/FeJ mouse right cranial lobe following E2Crimson *Mtb* WT infection over time.** (A) D28 post-infection (p.i.). (B) D42 p.i. (C) D70 p.i. Lesion formation during disease is depicted by the accumulation of myeloid cell around infecting *Mtb* (CD11b depicted in the colour gradient lookup table ‘flame’ and E2Crimson reporter for *Mtb* H37Rv WT shown in white). Lower panels depict ZY orthogonal positions in the lung stack, highlighting specific areas of interest (white box), showing merged signal of *Mtb* and CD11b. Scale bars: 1500 µm for the 3D panels, and 100 µm, 400 µm and 700 µm for the D28, D42 and D70 orthogonal sections, respectively. Images shown are from a single representative lung lobe of one mouse per group. Data in Fig. 4 represent the same lung lobes in the WT-infected groups. All lung lobes were imaged using uniform laser settings optimized for the weakest signal at D28 p.i. to ensure consistency across samples. This approach resulted in signal saturation at later time points, which was subsequently corrected by manually adjusting display thresholds in Imaris for visualization purposes.

We next utilized PACT-based clearing and whole-lung imaging of infected animals to track infection progression over time (28, 42 and 70 days post-infection) in 3D. To detect the majority of immune cells involved in the anti-TB response, we employed an anti-CD11b (also known as ITGAM) antibody (conjugated to near-infra-red fluorophores) to enable the identification of all myeloid cells (CD11b positive), coupled with the endogenous labelling of *Mtb* using the E2Crimson reporter. E2Crimson *Mtb* WT-infected C3HeB/FeJ mice showed early evidence of lesion formation at 28 days post-infection, with small foci of CD11b-positive cells stemming from an upper branch (Fig. 3A; Movie 2). Different from a classical single-plane visualization, the orthogonal view from the 3D stack of the lungs allows the visualization of whole-cell populations in defined tissue environments and *Mtb* growth (Fig. 3, XY projections; Movie 3), stemming from bronchioles in the ROIs. The proliferation of *Mtb* was apparent by day 42 post-infection (Fig. 3B; Movie 4) and highly evident by day 70 (Fig. 3C; Movie 5). Lesion size grew substantially by day 42 and showed evidence of merging by day 70. Comparison of fixed samples indicated that lesions appeared larger by day 42 and exhibited features that might be consistent with merging by day 70. Additionally, at day 70, we observed *Mtb* bacteria beyond the CD11b-positive cell clusters, potentially indicative of dissemination beyond the primary lesion (Fig. 3C). Although we acknowledge that these observations are from a single animal per timepoint, these results are further supported by the histological assessment (Fig. 1C).

**Fig. 4.**
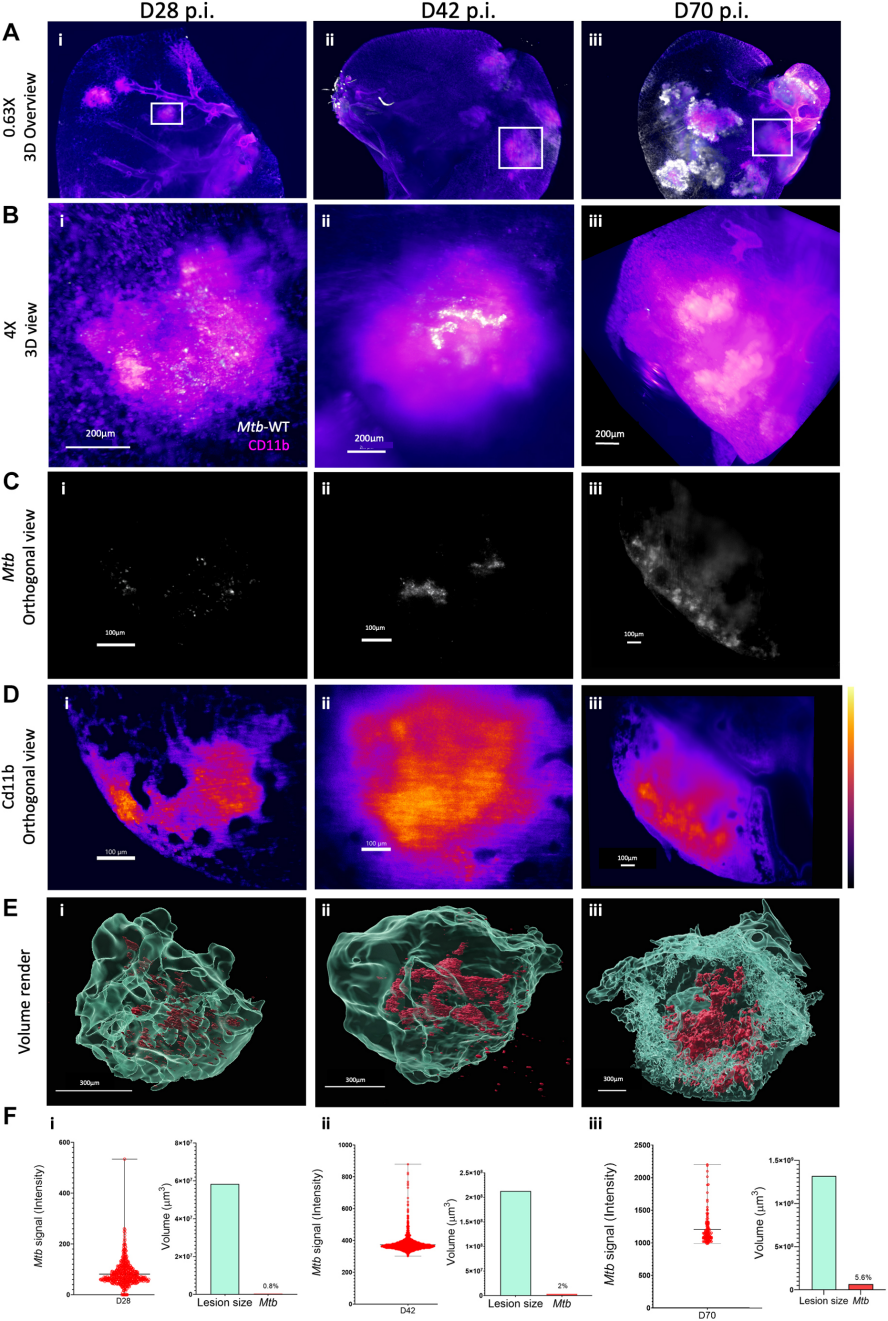
**Higher-resolution imaging of ROIs from the E2Crimson *Mtb* WT-infected group of C3HeB/FeJ mice.** Images are shown at D28 (i), D42 (ii) and D70 (iii) p.i. (A) ROIs identified in the larger stack under lower magnification (0.63×) are highlighted in white. Images represent the same samples shown in Fig. 3. (B) 3D projection of the ROI taken at higher resolution (4×, dynamic focus), showing the merged signal of *Mtb* (white) and CD11b (magenta)-positive cells in lesions at D28, D42 and D70. (C,D) Orthogonal view through the higher-resolution stack, showing the signal of *Mtb* (C) and CD11b (D)-positive cells (lookup table ‘flame’ display). (E) Volume render of the lesions, including render of the *Mtb*-infected cells. (F) Signal intensity from the E2Crimson reporter (left), and the volume size (µm^3^) of the lesion (cyan) and *Mtb* space (red) taken up inside the lesion (right). Bars represent the mean and maximum and minimum values.

We further imaged ROIs in the E2Crimson *Mtb* WT-infected group (Fig. 4A) at higher resolution using dynamic focusing (Fig. 4B) and quantified the volume of a CD11b-positive lesion, as well as the signal and infecting space taken up by *Mtb.* Orthogonal sections showed the location of *Mtb* (Fig. 4C, i-iii), and using the lookup table display allowed visualization of the density of CD11b-positive cells surrounding the infecting cells (Fig. 4D, i-iii). Volume rendering showed that the lesion grew substantially, from 5.84×10^7^ µm^3^ at day 28 (Fig. 4F, i) to 2.13×10^8^ µm^3^ at day 42 (Fig. 4F, ii) and 1.32×10^9^ µm^3^ by day 70 (Fig. 4F, iii). *Mtb* occupied 0.6% of the lesion space at day 28, reaching 2% and 5.6% by day 42 and day 70, respectively, with *Mtb* cells present outside the containment regions by these time points (Fig. 4E, ii and iii). Measuring signal intensity from the E2Crimson reporter indicated the expansion of *Mtb* growth over time (Fig. 4F, i-iii). Representative videos of the total lesion render for day 70 lung lobe are provided in Movie 6, and individual lesion and bacterial render in Movie 7.

### *Mtb* ESX-1 T7SS is required to shape lesion architecture

**Fig. 5.**
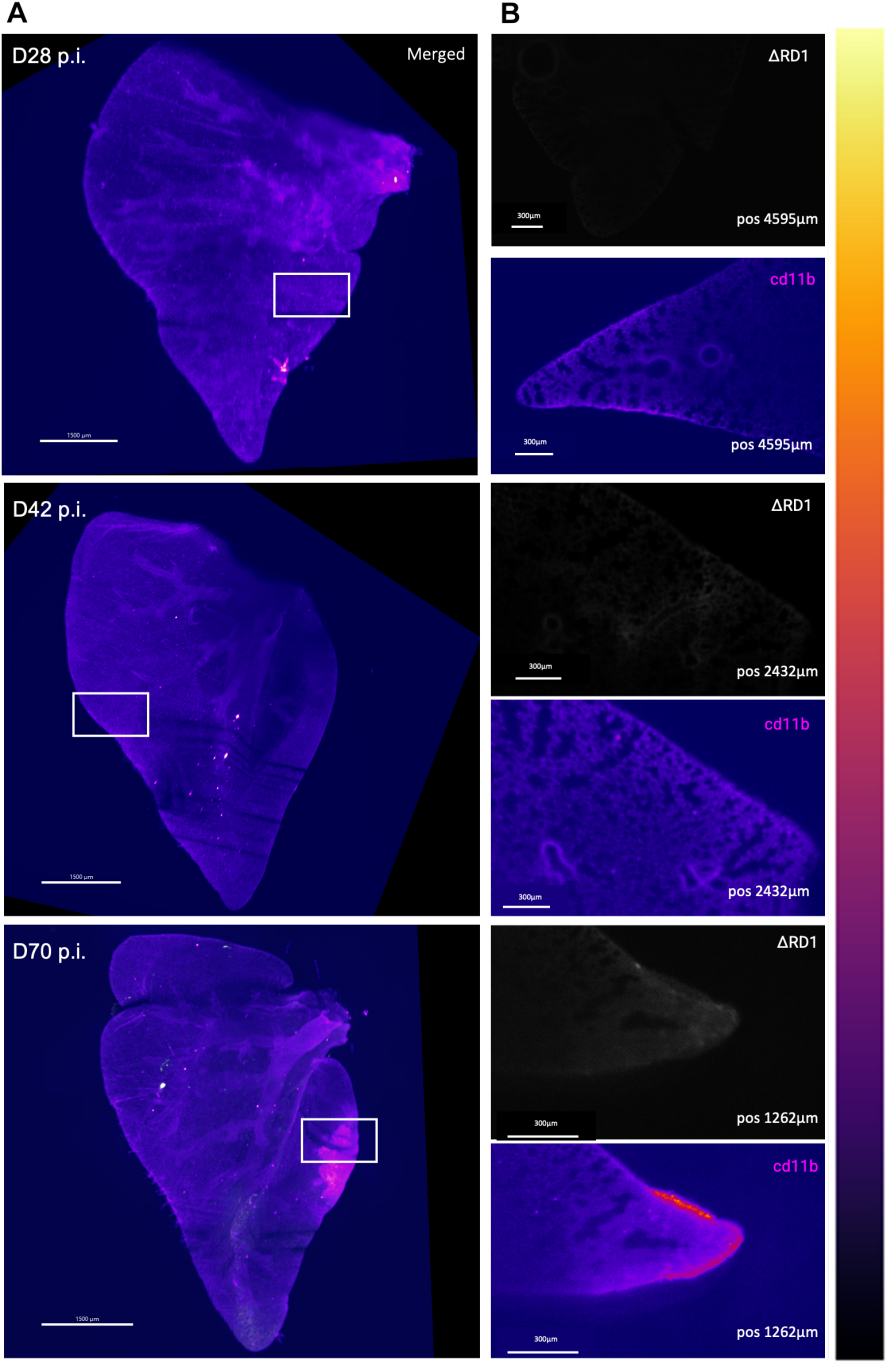
**LSFM-based 3D imaging of PACT-cleared C3HeB/Fej mouse lungs following infection with Mtb ΔRD1 mutant.** (A) 3D imaging of E2Crimson *Mtb* ΔRD1 infection over time (D28, D42 and D70 p.i.) in a C3HeB/FeJ mouse model, depicting lesion formation during disease progression [myeloid cell recruitment depicted in magenta (CD11b) and E2Crimson reporter for *Mtb* shown in the lookup table ‘fire’]. Images were acquired using identical laser and light-sheet settings for both *Mtb* WT (shown in Fig. 3) and *Mtb* ΔRD1 lung lobes. Brightness and contrast were adjusted to show visually similar intensities in the figure to account for excess intensity in the WT-infected group at later time points. (B) Zoomed orthogonal positions in the lung stack from the E2Crimson *Mtb* ΔRD1-infected lungs highlighting specific areas of interest (white box), showing signal of *Mtb* ΔRD1 (top) and CD11b (bottom).

We next performed whole-lobe imaging of E2Crimson *Mtb* ΔRD1-infected mice to compare the CD11b and *Mtb* spatio-temporal distribution pattern with that in the E2Crimson *Mtb* WT-infected mice (Wong, 2017). *Mtb* utilizes specialized secretion systems to exploit host responses with the ESX-1 secretion system encoded in the RD1 region of the genome that is critical to virulence in mice (Clemmensen et al., 2017; Cohen et al., 2018; Pan et al., 2005; Pym et al., 2002; Volkman et al., 2004; Wong, 2017). The 3D images are shown in Fig. 5A,B and Movie 8 (day 28), Movie 9 (day 42) and Movie 10 (day 70). LSFM imaging revealed that, in contrast to *Mtb* WT-infected mice, the E2Crimson *Mtb* ΔRD1-infected mice displayed diffuse signal of the *Mtb* mutant, only apparent at day 42 and day 70 post-infection. Moreover, the CD11b-enriched lesions showed disrupted, disorganized structures that were primarily present on the outer areas of the lung space with no formal structured lesion development visible (Fig. 5B).

**Fig. 6.**
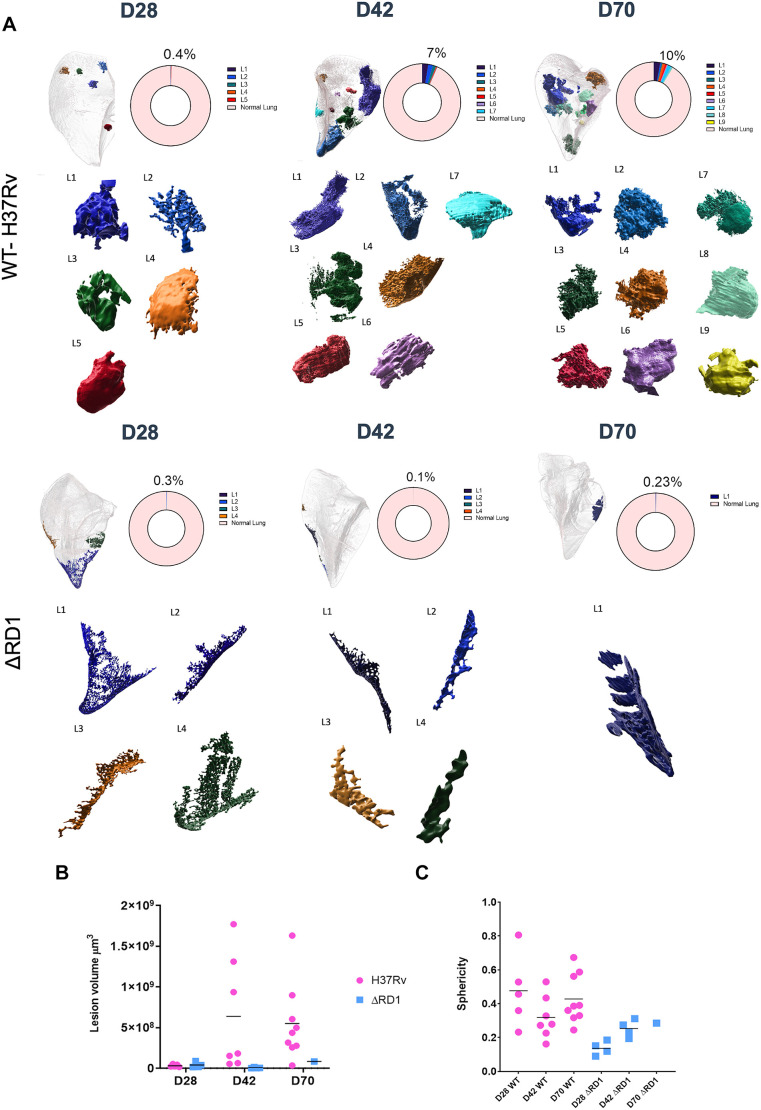
**Lesion size, distribution and sphericity within C3HeB/FeJ mouse lungs infected with *Mtb* H37Rv WT versus ΔRD1 infection over time.** (A) Lesion involvement calculated as a percentage of the total lung space, showing individual lesions rendered in 3D. Lesion size and sphericity were quantified using Imaris and the surface object detection and 3D volume rendering application, applying blend mode and manual intensity thresholding based on CD11b signal in each lung lobe for the lesion size, and autofluorescence signal to calculate the total lung volume. (B) Individual lesion volumes (µm^3^) plotted over the course of infection. The line indicates the median. (C) Sphericity of individual lesions in C3HeB/FeJ mice infected with different *Mtb* strains. Sphericity was calculated using Imaris and represents a dimensionless value ranging from 0 to 1, where 1 represents a perfect sphere. Sphericity provides insights into the compactness and shape characteristics of an object.

A quantitative analysis of lesion size and sphericity further revealed striking differences in development and progression of lesion architecture between the E2Crimson *Mtb* WT- and E2Crimson *Mtb* ΔRD1-infected lungs (Fig. 6). When normalized to healthy lung space, the volume of infected space taken up comprised 0.4% and 0.3% of the total lung volume at day 28 for the E2Crimson *Mtb* WT- and E2Crimson *Mtb* ΔRD1-infected mice, respectively (Fig. 6A). Lesion volume (Fig. 6B) at day 28 was similar for *Mtb* WT and *Mtb* ΔRD1 (mean volume of lesions=2.75×10^7^ µm^3^ versus 2.97×10^7^ µm^3^) but differed substantially by day 42 (2.96×10^8^ µm^3^ versus 3.08×10^6^ µm^3^) and day 70 (3.74×10^8^ µm^3^ versus 8.22×10^7^ µm^3^). Lung involvement did not change substantially for the mutant; however, in the E2Crimson *Mtb* WT-infected group, CD11b-positive lesions comprised 7% and 10% of the total lung space at day 42 and day 70, respectively. We used sphericity measurements to quantify the spatial organization of lesions, indicating spatially different lesions between the E2Crimson *Mtb* WT- and E2Crimson *Mtb* ΔRD1-infected mice (Fig. 6C). Altogether, using this approach we show that ESX-1 activity is required for lesion architecture in C3HeB/FeJ.

### Lesion ultrastructure through vCLEM shows that infection spreads from an initial macrophage population

**Fig. 7.**
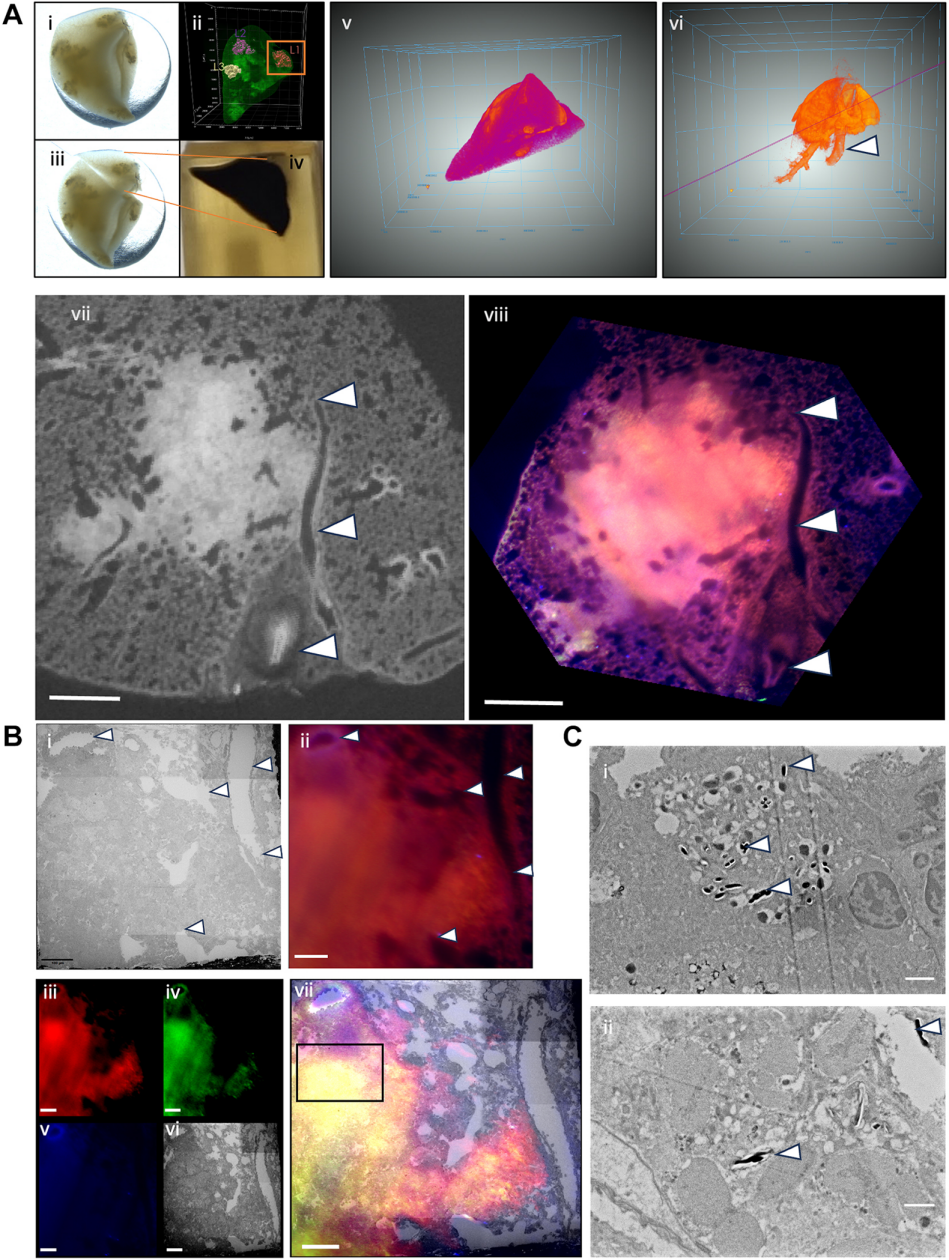
**Volumetric correlative light and electron microscopy (vCLEM) to interrogate PACT-cleared tissue for ultrastructural content imaging.** All images were taken from lung lobes at 42 days post-infection in both uncleared and cleared tissues. (A) Overview of region identification workflow. Entire lung lobe (i) with corresponding light-sheet render (ii) of each lesion of interest. Lung lobes were cut according to the localisation of each lesion (iii) and embedded in resin (iv). Care was taken to leave enough healthy surrounding tissue until fine block trimming could occur post resin embedding. Prior to block trimming, micro-computed tomography (microCT) scanning was performed on embedded tissue samples to assess the orientation of granulomas compared to initial light-sheet microscopy (v). Image registration between the microCT and light-sheet microscopy was conducted to aid in block trimming. Render of granuloma with lung tissue subtracted (vi). Purple line indicates the angle of the cross-section shown in vii. White arrowheads indicate corresponding blood vessels. Light-sheet data were transformed to microCT data using BigWarp (FIJI), to geometrically scale the light-sheet data to the same as those in the resin block (viii). An affine transformation was applied to the stack, with special care taken not to induce excessive deformation of the original image. The newly transformed light-sheet stack was then registered to the captured serial block face electron microscopy (SBF-EM) dataset, again using inherent morphological landmarks to identify appropriate areas for segmentation. Scale bars: 500 μm. (B) Use of the morphological landmarks to overlay fluorescent and SBF-EM datasets. Fine registration of SBF-EM (i) and light-sheet (ii) images using inherent morphological landmarks indicated by white arrowheads. Final channel overlay of the SBF-EM and the LSFM data (vii), indicated individually in the red (iii), green (iv) and far-red (v) channels post autofluorescence subtraction. Final electron microscopy overlay is shown in vi. Scale bars: 100 μm. The black box in vii represents the area further imaged by SBF-EM in Fig. 8. (C) Comparison of lung tissue before (i) and after (ii) clearing, showing scanning electron microscopy micrographs of 100 nm sections captured on silicon nano-wafers. Uncleared tissue was prepared with a conventional mega-metal staining protocol. Cleared tissue was prepared with ClearEM staining protocol. White arrowheads indicate *Mtb* bacteria within distinct vacuoles, indicative of engulfment by macrophages. Scale bars: 1 μm.

PACT clearing allowed for large-scale characterization of entire lung lobes and molecular identity through immunolabelling and LSFM. Given the abundance of E2Crimson signal at defined regions, we investigated whether there was a structural pattern to the observed clustering of the E2Crimson reporter at the ultrastructural level. Here, we opted to employ vCLEM to assess lesion ultrastructure in 3D at the subcellular level. This required the development of a gentle electron microscopy staining protocol (ClearEM), capable of preserving ultrastructural detail in cleared lung tissue. To confirm whether *Mtb* could still be observed post clearing, high-resolution scanning electron microscopy (SEM) was conducted on 100 nm sections from uncleared (Fig. 7C, i) and cleared (Fig. 7C, ii) *Mtb*-infected mouse lung samples. After confirming the presence of bacteria within cleared tissue samples, we proceeded with our vCLEM workflow, outlined in Fig. 7A. Following LSFM (Fig. 7A, ii), the lung lobes were embedded in resin (Fig. 7A, iv) and orientated using the LSFM data to further image using microCT (Fig. 7A, v) to determine block trimming for SBF-EM. Inherent morphological features were used as fiducial markers for multi-modal image registration and alignment. Airways and blood vessels could be used as landmarks to align the fluorescent data with the microCT datasets (Fig. 7A, vi) to allow for a final alignment overlay (Fig. 7B), as shown before with similar methods (Fearns et al., 2020).

**Fig. 8.**
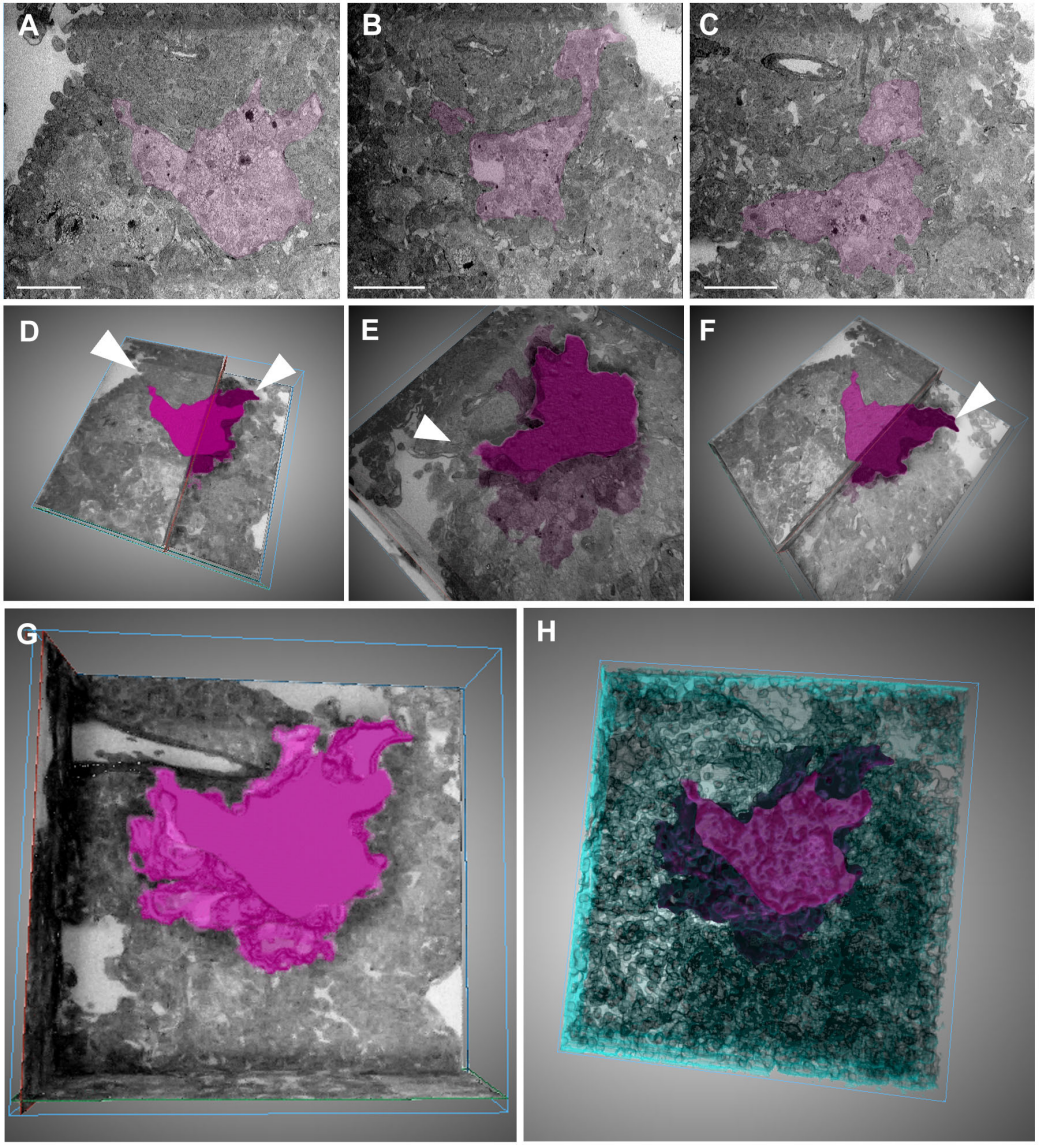
**Ultrastructural analysis and tracking of cell populations in infected lesions using SBF-EM.** (A-C) Region imaged using SBF-EM and the tracking of individual infected macrophage populations across multiple *z*-planes (black box in Fig. 7B, vii) at *z*=0 μm (A), *z*=300 μm (B) and *z*=600 μm (C). (D-F) Total volume render of infected macrophage population in A-C, respectively, within a selected lesion, with branch points from airways indicated by white arrowheads. (G,H) Render of infected population only (G), with the volume of surrounding structures shown in blue (H). Scale bars: 100 μm.

Tracking the cell population corresponding to the localization of the E2Crimson fluorescent signal revealed distinct clusters of cells that are less electron dense than the surrounding tissue (Fig. 8; Fig. S1). Furthermore, these regions extend from the surrounding airways, suggesting that the initial infection originates from macrophages recruited at airway-adjacent sites. Rather than forming a uniform structure, the lesion is organized into multiple, discrete compartments containing macrophage aggregates, as further illustrated in Movies 1 and 2. These observations provide novel insights into the spatial organization of granulomas, suggesting that distinct macrophage populations coexist within a single lesion and may differentially contribute to TB pathogenesis.

## DISCUSSION

Although classic light and fluorescence microscopy can provide insights into host–pathogen interactions, these methods are typically restricted to small, localized areas. As a result, histologic analysis often yields fragmented information and introduces sampling bias, limiting the ability to appreciate the overall anatomical context. In contrast, 3D imaging offers a powerful advantage, preserving tissue architecture and revealing key biological patterns that can identify clinically relevant features of TB pathology. Here, we developed an approach that combines the strengths of aqueous clearing (PACT), which preserves endogenous fluorescence while supporting versatile immunolabelling, with the rapid, large-scale imaging capabilities of LSFM and the resolution of SBF-EM. This multi-modal method enabled multi-scale imaging of TB infection within the C3HeB/FeJ mouse model, at multiple time points post-infection, bringing together molecular identity and ultrastructural context in a unique manner. Applying this approach enabled the visualization of significantly larger lung volumes, including entire murine lung lobes, which provides a major advantage for studying TB pathology. By capturing these large-scale, yet detailed, spatial relationships, this method enhances our ability to investigate the complexities of TB infection and treatment response in ways that were previously limited by smaller-scale imaging techniques.

Our results align with previous studies that have explored the 3D architecture of granuloma development during TB infection, including microCT, optical mesoscopy, ethyl cinnamate-based tissue clearing and PACT-based clearing (Cronan et al., 2015; Francis et al., 2020; Kang et al., 2020; Wells et al., 2021, 2022). MicroCT and volumetric imaging studies in humans have demonstrated that tuberculous granulomas exhibit complex, branched and cylindrical morphologies, intricately associated with the bronchial and vascular architecture. In parallel, the creation of high-resolution 3D atlases of resected human lung tissue during TB and coronavirus disease (COVID-19) infection has further emphasized the diverse architectural spectrum of granulomas, suggesting that the pathogen's genetic variations and host–tissue interactions play crucial roles in shaping these structures. Similarly, our study underscores the value of 3D imaging to broaden our understanding of pathogenesis and the clinical heterogeneity of TB.

Granuloma necrosis, a key pathogenic event, is heavily impacted by the ESX-1 secretion system, as extensively reported in the literature (Clemmensen et al., 2017; Driver et al., 2012; Kramnik, 2008; Pym et al., 2002; Stanley et al., 2007; Swaim et al., 2006; Wassermann et al., 2015). The development of necrotizing granulomas not only exacerbates disease severity but also precedes the formation of cavitary lesions, which are crucial for TB transmission. This process is particularly evident in C3HeB/FeJ mice, in which necrosis within granulomas is a distinct hallmark of *Mtb* infection (Pan et al., 2005). Given the established importance of ESX-1 in *Mtb* pathogenesis, we chose to utilize an ESX-1 deletion mutant in our study. This provided a solid foundation to investigate the key differences between virulent and attenuated strains during early and late stages of lesion formation. This well-defined model not only allows us to apply our novel 3D imaging methodology, but also facilitated the exploration of new insights into lesion architecture and dynamics.

Whole-lobe imaging allowed us to capture interesting events early in the infection, showcasing how ESX-1 virulence influences lesion formation. We noted that, by 28 days post-infection, lung involvement and lesion morphology differed significantly between WT- and RD1-infected mice, despite similar total CFU levels between the two bacterial strains at this timepoint. Early infection with virulent *Mtb* begins as small foci in the proximal airways, which subsequently disseminate to the lower regions of the lungs. We observed significant parenchymal disease progression and widespread dissemination of bacteria within the lung lobes. This aligns with previous studies that have shown that virulence factors drive early tissue destruction and enable infected macrophages to exit the primary granuloma, facilitating further spread of the infection (Driver et al., 2012; Kramnik, 2008; Pym et al., 2002; Volkman et al., 2004). By contrast, the RD1-infected mice showed that most lesions were located at the lung periphery and lacked any structured organization. The 3D rendering displayed very thin lesions characterized by minimal infiltration into the surrounding lung tissue. This morphology suggests a distinct growth pattern, in which the lesions extend without significant expansion into adjacent parenchyma, indicating limited host immune response and restricted tissue remodelling associated with the infection. As anticipated, the absence of organized lesions in ΔRD1-infected mice aligns with prior studies on the ESX-1 secretion system's role in promoting *Mtb* virulence and pathogenesis (Clemmensen et al., 2017; Pym et al., 2002; Stanley et al., 2007; Wassermann et al., 2015). Moreover, the 3D imaging of lesions across different infection stages and bacterial strains aligns with findings by Francis et al. (2020), who also noted strain-specific lesion pathology in murine models. Their comparison of H37Rv-infected BALB/c mice with Kenyan isolates showed greater lesion volume and frequency in the H37Rv-infected mice (Francis et al., 2020). This cumulative evidence suggests that whole-lobe, high-resolution imaging approaches, such as those in our study, can offer crucial insights into strain-specific disease progression and immune response within complex tissue architectures. Although our study primarily focused on lesion architecture and cellular organization, future research with targeted cell death markers could enhance understanding of necrosis and cell turnover during infection. Such approaches would offer a unique view of early granuloma necrosis and its progression in relation to ESX-1 activity.

Our study showcases the application of PACT-based clearing with LSFM and SBF-EM to investigate lesion architecture and progression; however, several limitations warrant discussion to fully interpret our findings and guide future research. Although we were limited by the available fluorescent channels, LSFM imaging allowed faster and deeper imaging than can be achieved with standard confocal microscopy and without the requirement for sectioning of the tissue. This, however, came at a resolution cost because the long working distance of the objective lens limits spatial resolution that can be achieved for an entire lung lobe. Nevertheless, we showed that higher-resolution imaging of ROIs can be achieved through higher magnification [6.3× zoom; 0.5 numerical aperture (NA)], together with dynamic focus mode and thin light sheet, although this comes at a cost in acquisition time. A higher NA allowed the visualization of individual *Mtb* cells in the orthogonal view of specific lesions, although most of the *Mtb* signal is difficult to resolve as single cells, specifically at later time points. The resolution achieved is in line with a study that utilized PACT-based clearing to image a whole lung lobe infected with a tdTomato-expressing *Mtb* (Cronan et al., 2015), as well as a study that utilized mesoscopy and CUBIC Acid-Fast staining (Francis et al., 2020). In this study, the authors were able to achieve cellular resolution of *Mtb* using Mesolens (4×/0.47 NA) and Auramine and Rhodamine B staining (Francis et al., 2020). Although signal intensity from dyes can be used as a general marker of proliferation, the advancements of metabolic probes for *Mtb* (MacGilvary and Tan, 2018) could be utilized in future studies to provide increased information on the state of the pathogen in different infecting zones. The extensive infiltration of CD11b-positive cells within the lesions, coupled with the inherent limitations in spatial resolution of our imaging technique, prevented accurate quantification of cell numbers in this study. Although it could be hypothesized that the total volume of the cellular aggregates correlates with the number of infiltrating cells, further investigations would be necessary to derive precise cell density metrics, potentially employing techniques such as laser scanning microscopy in smaller sections of tissue. Our choice of imaging modality was based on achieving an optimal balance between thorough volume analysis and resolution sufficient for identifying cellular clusters. This observation aligns with the findings reported by Kang et al. (2020), which similarly noted the presence of large cell aggregates that posed challenges in resolution. Although the study by Kang et al. (2020) endeavoured to estimate cell counts by normalizing fluorescent reporter signals to the lesion volume, accurate quantification was ultimately achieved through flow cytometric analysis.

As stated, LSFM enabled whole-lobe imaging, yet the level of resolution at the cellular level was compromised. We wanted to investigate whether volumetric electron microscopy could be used to further reveal ultrastructural detail in PACT-cleared tissue, which has not previously been described. Although limited to a single granuloma, we were able to show that clarified tissue can be further utilized for ultrastructural imaging, albeit with a loss of membrane integrity. To our knowledge, this is the first time PACT-based clearing and SBF-EM have been combined in a single study. Manual rendering allowed us to identify not only the spatial organization of macrophage aggregates in detail, but also to visualize their distribution relative to the airways at multiple time points. Future work will focus on refining the clearing and embedding protocols to minimize membrane damage and further improve image quality. By optimizing staining procedures, reducing tissue handling steps, and exploring alternative embedding resins or fixation methods, it may be possible to achieve crisper images and more stable ultrastructural preservation. Although these refinements are still needed, the current study represents a significant leap forward, demonstrating the feasibility of interrogating cleared tissues by SBF-EM and paving the way for more detailed, high-resolution analyses of host–pathogen interactions in the future.

One of the main limitations of this study is the laborious and time-consuming procedures required to prepare samples, which make it impractical to assess large number of animals at one time. Immunolabelling takes an extremely long time to reach the tissue and requires large amounts of antibody. Although methods to speed up this process have been proposed, such as ACT-PRESTO (Lee et al., 2016), we did not find this to work in our experimental setup. We propose that transgenic mice that express fluorescent reporters in cells of interest would be a more practical method to visualize and circumvent lengthy antibody incubation times. Future studies that emphasize a broader immune profiling approach would provide richer and more compelling insights into the dynamics of different myeloid populations and lymphocytes throughout the course of infection.

Finally, the use of post-mortem lung tissue samples precludes the dynamic assessment of lesion heterogeneity and development within individual animals over time. Although this approach provides invaluable insights into the overall progression of lung pathology during TB infection, it does not capture the subtle changes in individual lesion characteristics that can occur within a host as the disease evolves. Longitudinal studies with serial non-invasive imaging or biopsy would be necessary to fully characterize these temporal dynamics (Ordonez et al., 2021). Further, as stated earlier, the extended time for tissue clearing, labelling and imaging limited our analysis to a single lung lobe per time point. As a result, although our observations are intriguing, additional data are needed to determine whether the differences are biologically significant as we were only able to image a single lung from an infected mouse per time point.

Altogether, we have developed a novel approach that combines PACT clearing, LSFM imaging and SBF-EM to provide a comprehensive and unbiased method for investigating TB infection in 3D. This multi-modal approach allows the visualization of entire infected lung regions and the identification of critical pathological features across different *Mtb* virulence conditions. These results further highlight the importance of considering the 3D spatial relationships within lung tissue to better understand the pathophysiology of tuberculosis and optimize treatment approaches for TB.

## MATERIALS AND METHODS

### Mice

Research protocols adhered to guidelines set out in the South African National Standard 10386 (2021) and were approved by Stellenbosch University Research Ethics Committee Animal Care and Use. All procedures were conducted according to the Veterinary and Paraveterinary Professions Act (Act 19 of 1982) and the Animal Diseases Act (Act 35 of 1984). Specific pathogen-free C3HeB/FeJ mice were purchased from The Jackson Laboratory (Bar Harbor, ME, USA). Female mice (8-10 weeks of age) were housed at five mice per cage in individually ventilated microisolator cages under biosafety level III containment under sterile conditions with sterile bedding, water and standard mouse chow. Infected mice were monitored daily for welfare and weighed every second week.

### Bacterial strains

The laboratory strain of *M. tuberculosis* (H37Rv) and an ESX-1 deletion mutant (ΔRD1), both transformed with a PTEC-19 plasmid (episomal) containing an E2Crimson fluorescent reporter and hygromycin resistance cassette, were used for aerosol infections. Bacteria were grown in Middlebrook 7H9 medium containing 0.05% Tween 20 and supplemented with 10% OADC (oleic acid, albumin, dextrose and catalase; BD Biosciences) and 50 μg/μl Hygromycin B (Gibco™, Thermo Fisher Scientific) in vented flasks and kept stationary at 37°C until mid-exponential phase was reached (optical density at 600 nm, 0.6-1.0) and enumerated by colony counting on 7H11 agar plates (described below). Culture stocks were stored at −80°C until use.

### Aerosol infection

Eight- to 10-week-old mice (40/infecting strain) were infected utilizing an inhalation exposure system (Glas-Col, Terre Haute, IN, USA). Bacterial strains from frozen stocks (H37Rv-WT and ΔRD1 mutant) were thawed and suspended repetitively using a Luer lock syringe attached to a 26 Ga hypodermic needle to obtain a single-cell suspension. Infection inocula were prepared to a final concentration of 2×10^6^ CFU/ml in autoclaved deionized water, and 5 ml of this inoculum was placed in the Glas-Col nebulizer. The infection procedures included 10 compressed air and 60 main (negative) standard cubic feet per hour (SCFH); 15 min preheat time; 30 min nebulizing time; 45 min cloud decay time; and 15 min decontamination time. Enumeration of the bacterial inoculum for both strains was determined by CFU counts on 7H11 plates, and the actual bacterial load delivered to the animals was confirmed from ten mice/strain 1 day post-aerosol challenge in total lung homogenate.

### Necropsy and organ harvesting

Following euthanasia using inhalation anaesthetic overdose, mice were individually transcardially perfused using 10 ml heparinized saline solution until lungs inflated and turned white, indicating red blood cell removal. Whole left lung lobes were removed and placed in 5 ml sterile PBS for enumeration of bacterial load. Right cranial and middle lobes were placed in 4% PBS-buffered paraformaldehyde, and caudal lobes were placed in 10% formalin for formalin-fixed paraffin embedding and histology.

### Enumeration of bacterial load of lungs

Lung lobes were disrupted in 5 ml sterile PBS containing 1.4 mm ceramic (zirconium oxide) beads (Bertin Instruments, Bertin Technologies, Montigny-le-Bretonneux, France) using a tissue homogenizer (Precellys Evolution, Bertin Instruments), serially diluted and plated onto Middlebrook 7H11 agar plates supplemented with OADC. Colonies were counted after 21 days incubation at 37°C. Bacterial counts were log_10_ transformed and expressed as mean log10 CFU±s.e.m. for each group.

### Pathology of lungs

Right caudal lobes were embedded in paraffin and cut to 5 μm thickness using a microtome before mounting onto glass slides. Sections were deparaffinized and stained with Haemotoxylin and Eosin for standard pathological assessment. Slides were scanned on a Ocus® 40 scanner (Grundium, Tampere, Finland) with a UplanXApo 20× objective (0.75 NA; resolution, 10 µm/pixel; image sensor, 12 Mpixel). Analysis of histology slides from each timepoint was done using QuPath v0.3.2 open-source software (Bankhead et al., 2017). This software was used to quantify the percentage lung involvement of lesions following infection with *Mtb* WT or ΔRD1 mutant. For each image used in the analysis, the magic brush tool was used to annotate the respective section of the image. A separate copy of the image was made in which only lung lesions, if present, were annotated used the same method. Specific adjustments were made for each annotation for each image to match the anatomy of each section and for consistency. The QuPath cell detection function was used to quantify and mark cells within an annotated area.

For each annotation, the number of detections and area were exported and analysed. Analysis of percentage lung involvement was completed by dividing the number of cell detections in lesions by the overall number of cell detections and then multiplying by 100. One-way ANOVA was performed with a Kruskal–Wallis post-test. Graphs presents the results as mean±s.e.m.

### Tissue clarification

Paraformaldehyde-fixed samples were transferred to freshly prepared hydrogel monomer solution (A4P4, 4% acrylamide (Sigma-Aldrich) in 0.1 M PBS) containing 0.25% photoinitiator VA-044 (Wako Chemicals) and incubated at 4°C for 48 h to allow permeation into the tissues. Samples were degassed for 20 min by bubbling nitrogen gas in the medium and placed at 37°C for 4 h for embedding. Following polymerization of the monomer, lungs were carefully removed from the hydrogel, rinsed in 0.1 M PBS and transferred to clearing solution [8% sodium dodecyl sulfate (SDS), Sigma-Aldrich] in 0.1 M PBS containing 50 mM sodium sulfite (Sigma-Aldrich), pH 8.5. Samples were incubated in the dark at 37°C with gentle shaking (100 rpm), and clearing solution was replaced daily. Once optical transparency was achieved (∼4 days), SDS was washed out with sequential wash steps in wash buffer (0.1% Triton X-100 in 0.1 M PBS containing 50 mM sodium sulfite, pH 8.5) by diluting the clearing solution 1:1 with pre-warmed wash buffer three times daily for 2 days, gradually reducing the temperature until room temperature was reached. Samples were stored in 0.1 M PBS containing 0.01% sodium azide before immunostaining and imaging.

### Immunostaining

Lung lobes were washed twice for 20 min using 0.1 M PBS and transferred into 10 ml/lobe blocking buffer (3% bovine serum albumin in 0.1 M PBS containing 0.1% Triton X-100 and 0.01% sodium azide) and incubated at 37°C with gentle shaking for 2 days in the dark. Lung lobes were stained for a total period of 5 days, progressively increasing antibody concentration every day, reaching final concentration of 1:50 by day 4. This was done to prevent antibody accumulation on the outside of the lungs, which was observed during optimization. A total volume of 3 ml primary antibody (1:50; anti-CD11b; PA5-79532, Invitrogen) in blocking buffer was used to stain each lung lobe at 37°C with gentle shaking for 4 days in the dark. Following immunostaining, lung lobes were washed for 3 days in 50 ml 0.1 M PBS containing 0.1% Triton X-100 at room temperature, changing the wash solution twice daily. Lung lobes were then transferred to secondary antibody solution (1:50; Alexa Fluor 790 AffiniPure Fab Fragment Goat Anti-Rabbit, 111-657-008, Jackson ImmunoResearch), in blocking buffer and incubated for a further 2 days at 37°C with gentle shaking for in the dark. A further 2 days of washing in 0.1 M PBS containing 0.1% Triton X-100 was conducted to wash off unbound secondary antibody. All blocking buffer solutions were filter sterilized using a 0.22 µm filter (Sigma-Aldrich).

### RIMS matching

Refractive index (RI) matching (Yang et al., 2014) was done by immersing the stained lung lobes in home-made RIMS at an RI of 1.45. Briefly, 143.78 g HistoDenz™ (Sigma-Aldrich) was dissolved in 0.02 M PB buffer containing 0.1% Tween 20 and 0.01% sodium azide; pH was adjusted to 7.5. A refractometer was used to confirm the RI of the solution. Lung lobes were incubated in excess RIMS solution (5 ml) in the dark until completely transparent. The same solution was used to fill the imaging chamber during imaging.

### LSFM and data acquisition

Lung lobes were mounted directly onto platforms with either screws or spikes to fix the sample in place, ensuring that the sample was stable throughout acquisition. Sample orientation was carefully chosen to ensure that propagation length of the excitation light is a short as possible, while maintaining that the full ROI does not exceed the working distance of the objective. The chamber was filled with 130 ml of the RIMS matching solution. Single-plane illuminated (light-sheet) image stacks of cleared lung lobes were acquired using a LaVision Biotec Ultramicroscope II Light Sheet system (Miltenyi Biotec GmbH, Bergisch Gladbach, Germany) equipped with Olympus MVX-10 Illumination microscope body and MV PLAPO 2XC objective. Samples were illuminated by three light sheets from the left and right side. Whole lung lobes were acquired at 0.63× zoom with an exposure time of 100 ms, *z*-stack intervals of 6 µm and light-sheet thickness of 12 µm. The *Mtb* E2Crimson reporter was imaged with a 640 nm laser line and 680/30 nm emission filter with laser power at 90%, and CD11b signal was imaged using a 784 nm laser line and 835/70 nm emission filter with laser power of 100%. Where autofluorescent signal was required, green signal was imaged using the 488 nm laser (excitation) and 525/50 nm emission filter with 20% laser power. Higher-resolution images were taken on areas of interest using either the 4× or 6.3× zoom, depending on where the lesion was situated and the working distance available. Dynamic focus allowed capturing of high-resolution images across a large area. Instead of keeping the light sheet in one location and moving the sample to tile the image, dynamic focusing keeps the sample stationary and adjusts the horizontal focus position of the sheet throughout it. Multiple images are acquired and then blended to ensure maximal *Z* resolution across the entire field of view. Dynamic horizontal focusing mode was employed with blending mode set to the centre of the image, with light-sheet thickness adjusted to 3.8 µm and a step size of 0.5 µm. ImSpector Pro software (Abberior Instruments, Göttingen, Germany) was used for imaging and Imaris software v9.7.2 (Bitplane, Oxford Instruments, Zurich, Switzerland) was employed to analyse LSFM images. Image stacks were converted to native ims. files using file converter tool and used to generate 3D video files. The surface creation tool was used to create a ROI to set threshold signals, after which the entire image was processed. In the surface creation wizard, the source channel for either the E2Crimson reporter or AF790 channel was selected. Smoothing surface detail was set to 7 µm. Maximum intensity was used to identify the cell of interest in each lobe, and number of voxels was kept at 1 mg and gamma as 1.00. Adjustments to surface shape and detection were made manually to reduce extraneous noise, particularly where the signal was oversaturated in the later time points. Spot counting was used using the Imaris spot component, segmenting ROI for analysis and using ‘different spot size’ feature to assign different sizes based on the adjusted signals. Estimated XY diameter was assigned as 5 µm, and background subtraction (Gaussian) was enabled. Model point spread function elongation along the *z*-axis was enabled to created elliptical shaped spots. Threshold was adjusted manually to have as many single spots as possible per identification. Threshold signals were set as follows (in pixels): H37Rv WT: day 28=maximum (max) 2102, minimum (min) 511; day 42=max 2160, min 511; day 70=max 1241, min 241; and RD1 mutant: day 28=max 4849, min 249, day 42=max 6697, min 249, day 70=max 2551, min 249. Zoomed lesions were analysed similarly, with threshold settings as follows (in pixels): day 28=max 534, min 0; day 42=max 879, min 302, day 70=max 7969, min 987. Individual lesion size, volume involvement and sphericity were calculated using Imaris software and the surface object detection and 3D volume rendering tools with blend mode, with manual thresholding of intensity based on individual CD11b signal in each lobe to differentiate the object of interest from the background and Gaussian smoothing of 2 µm. The volume of the object was calculated by summing the volumes of all the voxels that lie within the boundary of the segmented surface. Volume of a single voxel=voxel size in *x*-dimension×voxel size in *y*-dimension×voxel size in *z*-dimension (this gives the physical volume of each voxel in μm^3^ or other units). Similarly, the volume of the total lung was calculated using the surface object tool and the autofluorescent channel to capture the full lung space instead. Percentage lung involvement was then calculated as volume of the total lesions/total lung volume and expressed as a percentage. Sphericity was calculated on the rendered lesions by applying the sphericity tool in Imaris; briefly, this is a measure of how closely the shape of a 3D object resembles a perfect sphere. It is a dimensionless value ranging from 0 to 1, where 1 represents a perfect sphere. Sphericity provides insights into the compactness and shape characteristics of an object.

Sphericity (Ψ) is calculated using the following formula:
()

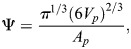
where ‘*V*_*p*_’ represents the volume of the particle and ‘*A*_*p*_’ represents the surface area of the particle.

### vCLEM

#### Sample preparation for SBF-EM

Following light-sheet microscopy, lung lobes were cut according to the localization of each lesion. Care was taken to leave enough healthy surrounding tissue until fine block trimming could occur post resin embedding. Thereafter, samples were fixed for 24 h at 4°C with a mixture of 2.5% glutaraldehyde and 4% formaldehyde in 0.1 M phosphate buffer. Thereafter, incubation with 2% reduced osmium tetroxide (OsO_4_) (mixture of 4% OsO_4_ and 3% potassium ferricyanide, 1:1) was conducted for 60 min on ice, followed by 20 min incubation with thiocarbohydrazide at room temperature, 30 min incubation with aqueous osmium tetroxide at room temperature and overnight incubation with 1% uranyl acetate at 4°C. Between each incubation step, samples were washed three times with distilled water (dH_2_O).

After overnight incubation, dehydration in a graded ethanol series of increasing concentrations was conducted for 10 min each on ice (20%, 50%, 70%, 90%, 100%), followed by two rounds of 100% ethanol dehydration at room temperature. Resin infiltration was performed in a series of 2 h incubation steps at room temperature, using increasing concentrations of EPON resin diluted in acetonitrile (25%, 50% and 75%). A final infiltration step with 100% EPON resin was performed overnight on a rotator, followed by incubation with fresh resin for 4 h the next day. Lastly, samples were transferred to embedding mounts and placed in an oven at 60°C for 2 days to allow for appropriate polymerization and hardening of resin.

#### MicroCT scanning

Prior to block trimming, microCT scanning was performed on embedded tissue samples to assess the orientation of granulomas compared to initial light-sheet microscopy, as described previously (Fearns et al., 2020).

#### Region finding and block trimming

Resin blocks were then trimmed down coarsely by hand, followed by fine trimming of an initial ROI spanning 2×2×1 mm using a glass knife and UC7 ultramicrotome (Leica Microsystems). The trimmed block was then mounted on an ultramicrotome stub using conductive silver epoxy (S-05000-AB, SPI, West Chester, PA, USA). To facilitate hardening of the silver epoxy, samples were transferred to an oven for 2 h at 60°C. Excess epoxy was trimmed away, and fine cutting of the resin block continued with the ultramicrotome, this time using a 45° diamond knife (Diatome, Quakertown, PA, USA). When the block face reached an approximate area of 0.7×0.7 mm, 100 nm thin sections were captured on silicon nano-wafers to assess tissue ultrastructure through SEM (Apreo SEM, Thermo Fisher Scientific). Once the ROI was confirmed to be a granuloma, with corresponding morphological landmarks to that of the microCT data, the block face was sputter coated with 50 nm gold/palladium (Denton Desk V, Denton Vacuum, Moorestown, NJ, USA) followed by another round of ultramicrotome sectioning (80 nm thin sections) to expose the sample block face.

The sample stub was placed into the ultramicrotome attachment of the Apreo Volumescope (Thermo Fisher Scientific), and eucentric calibration of the diamond knife to the block face was conducted. Image acquisition was conducted at an adjusted chamber pressure of 0.5 mbar to compensate for excessive charging of the resin. Under a lowered chamber vacuum pressure of 0.5 mbar, the VolumeScope Dual Back Scatter (VS-DBS) detector was implemented, using a voltage of 2.00 kV and probe current of 0.20 nA. Region finding and beam energy alignment were conducted using Xt Microscopy and Maps 3.9 software (Thermo Fisher Scientific). A *z*-width of 100 nm was deemed appropriate as a cutting thickness. Serial block-face data were acquired for a total of 2000 slices using the VS-DBS detector at a pixel resolution of 3840×2160 for each tile, with a scan speed of 2 µs.

#### Image processing

After acquisition, tiled *z*-stacks were stitched using the MIST plugin in FIJI (ImageJ) followed by SIFT linear alignment of *z*-stacks in FIJI (ImageJ). Image processing involved applying a 2D gaussian blur (sigma=2), recursive exponential filter (3×3×3 pixels) and a final 3D denoising filter (5×5×5 pixels) in Amria (2019.3) (Thermo Fisher Scientific).

Image registration between the three modalities involved transforming light-sheet data to microCT image stacks using BigWarp (FIJI), to geometrically scale the light-sheet data to the same as those in the resin block. An affine transformation was applied to the stack, with special care taken not to induce excessive deformation of the original image. Given that a subsequent transformation would be necessary for alignment to the SBF-EM data, a similar transformation was deemed unfit. The newly transformed light-sheet stack was then registered to the captured SBF-EM dataset, again using inherent morphological landmarks in order to identify appropriate areas for segmentation.

Once identified, manual segmentation was conducted on ROIs using ORS Dragonfly (2022.1) (Comet, Montreal, Quebec), with all subsequent renders and image panels also created in the same software.

### Statistical analysis

All statistical analysis was performed using GraphPad Prism version 7. Specific tests employed are described in figure legends.

## Supplementary Material

10.1242/dmm.052185_sup1 Supplementary information
