# Supplementary material for: Correlative 3D imaging method for analysing lesion architecture in susceptible mice infected with Mycobacterium tuberculosis
Source: Dis Model Mech. 2025 Mar 26;18(9):dmm052185. doi: 10.1242/dmm.052185 (PMC11972079; doi:10.1242/dmm.052185)
Supplement: Supplementary information [file dmm-18-052185-s1.pdf]

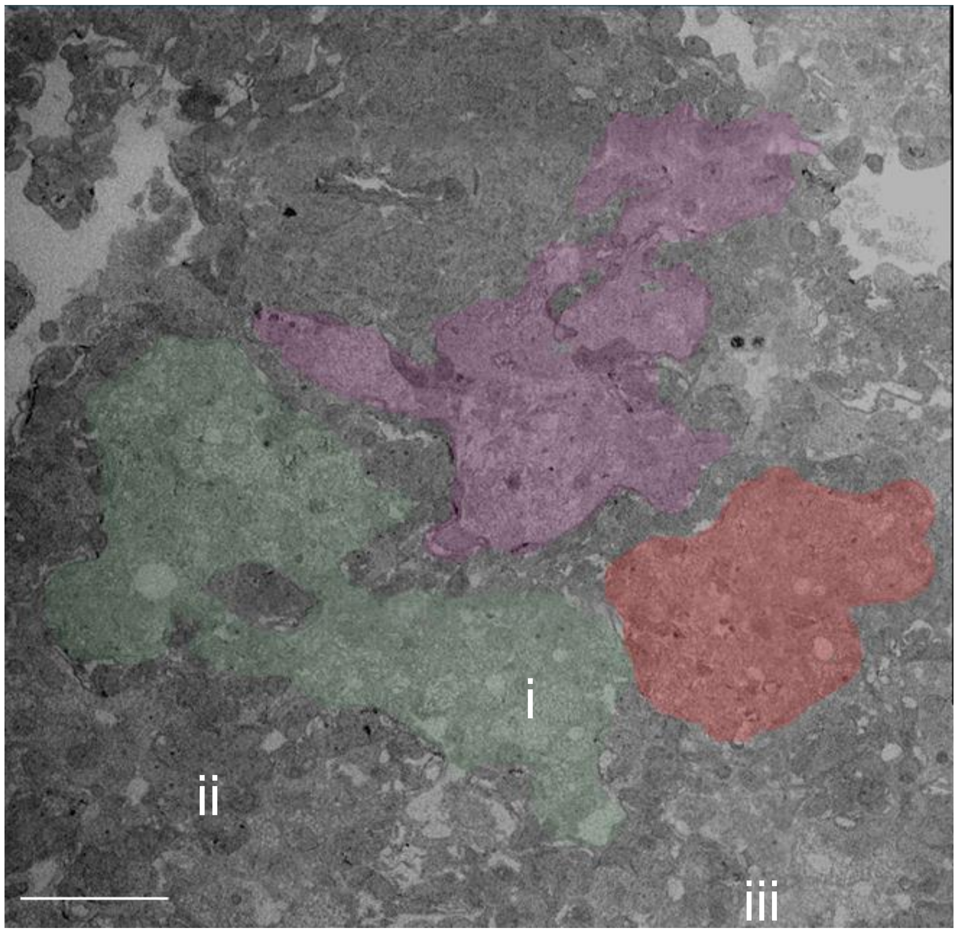

Fig. S1. Individual z-slice in SBF-SEM z-stack indicating multiple sub-lesion areas within one positively identified lesion (i, ii and iii). Scale bar 100um.

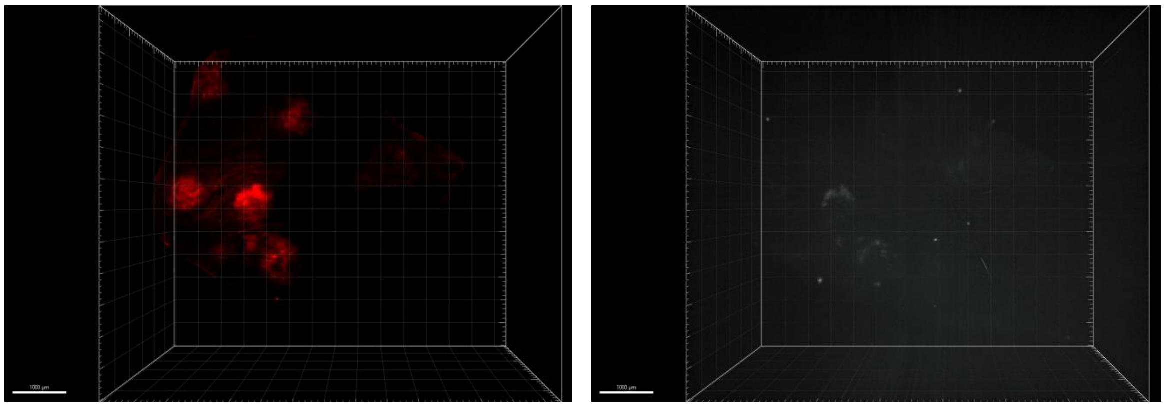

Fig. S2. Channel bleed through assessment of the E2 Crimson reporter. Whole lung lobe infected with E2 Crimson Mtb at D42 post-infection stained with AF790 secondary antibody only and imaged under 647 and 790 laser lines to establish specificity of the E2 Crimson reporter.

Disease Models & Mechanisms • Supplementary information

Table S1. Pathology assessment

H37RV-WT DAY28

**Image**	73_22 Mouse A_28 H37 Rv.svs	74_22 Mouse B_28 H37 Rv.svs	75_22 Mouse C_28 H37 Rv.svs	76_22 Mouse D_28 H37 Rv.svs	77_22 Mouse E_28 H37 Rv.svs	78_22 Mouse F_28 H37 Rv.svs	79_22 Mouse G_28 H37 Rv.svs	80_22 Mouse H_28 H37 Rv.svs	81_22 Mouse I_28 H37 Rv.svs	82_22 Mouse J_28 H37 Rv.svs
**Name**	PathAnnotationObject	PathAnnotationObject	PathAnnotationObject	PathAnnotationObject	PathAnnotationObject	PathAnnotationObject	PathAnnotationObject	PathAnnotationObject	PathAnnotationObject	PathAnnotationObject
**Class**	null	null	null	null	null	null	null	null	null	null
**Parent**	Image	Image	Image	Image	Image	Image	Image	Image	Image	Image
**ROI**	Geometry	Geometry	Geometry	Polygon	Geometry	Geometry	Geometry	Polygon	Geometry	Geometry
**Centroid X µm**	8504,424	27759,5389	5216,6762	5456,2719	5223,9691	5364,5488	5421,542	5287,3749	5129,8159	26311,1985
**Centroid Y µm**	4099,0433	5777,8471	4137,853	4523,919	4352,1333	4520,8301	6506,3617	4705,1013	8247,1564	5160,8357
**Num Detections**	64752	49108	49167	22598	49538	47844	40797	41172	55858	28030
**Area µm^2**	15646667,53	14872673,39	15372142,81	7497411,865	11376292,31	13098179,1	10863615,03	9399329,179	12285728,19	8362048,202
**Perimeter µm**	37882,3463	21453,1024	21792,6996	18907,7268	24807,6976	21568,9579	22223,1388	24562,3839	26008,0645	35279,6018
**Image**	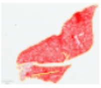	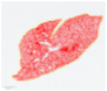	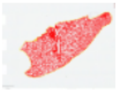	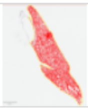	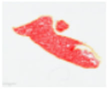	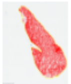	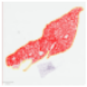	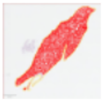	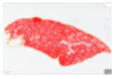	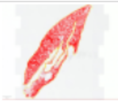
**Cells per um^2**	0,004138389	0,003301895	0,003198448	0,003014107	0,004354494	0,003652721	0,00375538	0,004380313	0,004546576	0,00335205
**% cells per um^2**	0,413838921	0,33018946	0,319844804	0,301410679	0,43544943	0,365272147	0,375537976	0,43803126	0,454657625	0,335204956
**Lesion detection**										
**Image**	73_22 Mouse A_28 H37 Rv.svs	74_22 Mouse B_28 H37 Rv.svs	75_22 Mouse C_28 H37 Rv.svs	76_22 Mouse D_28 H37 Rv.svs	77_22 Mouse E_28 H37 Rv.svs	78_22 Mouse F_28 H37 Rv.svs	79_22 Mouse G_28 H37 Rv.svs	80_22 Mouse H_28 H37 Rv.svs	81_22 Mouse I_28 H37 Rv.svs	82_22 Mouse J_28 H37 Rv.svs
**Name**	PathAnnotationObject	PathAnnotationObject	PathAnnotationObject	PathAnnotationObject	PathAnnotationObject	PathAnnotationObject	PathAnnotationObject	PathAnnotationObject	PathAnnotationObject	PathAnnotationObject
**Class**	null	null	null	null	null	null	null	null	null	null
**Parent**	Image	Image	Image	Image	Image	Image	Image	Image	Image	Image
**ROI**	Geometry	Geometry	Geometry	Geometry	Geometry	Geometry	Geometry	Geometry	Polygon	Geometry
**Centroid X µm**	9947,1552	5107,5578	5578,0969	5176,5624	6069,1235	6743,9927	4799,2032	7102,974	3777,3731	2535,7666
**Centroid Y µm**	2093,3996	3847,008	3224,8998	3175,8494	4331,4652	4967,9274	7115,554	3544,8851	6946,2077	4175,3962
**Num Detections**	4551	1126	3921	962	1516	1797	243	644	1625	626
**Area µm^2**	413838,3535	94521,8848	374227,4842	98244,3934	103474,3465	159652,3327	25433,6975	61273,7442	164787,6743	42402,3805
**Perimeter µm**	4590,7252	2675,9304	5380,8586	3026,4685	3534,3245	4072,7166	1285,4134	2178,2538	2428,2925	1991,2276
**Image**	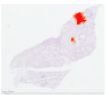	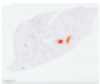	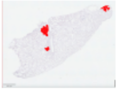	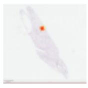	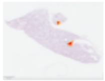		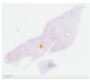			
**rea lung involvem**	2,644897725	0,635540648	2,434452299	1,310377436	0,90956125	1,218889522	0,234118177	0,651894864	1,341293506	0,507081273

Disease Models & Mechanisms • Supplementary information

H37RV-RD1 DAY28

**Image**	63_22 Mouse A_D28 RD1.svs	64_22 Mouse B_D28 RD1.svs	65_22 Mouse C_D28 RD1.svs	66_22 Mouse D_D28 RD1.svs	67_22 Mouse E_D28 RD1.svs	68_22 Mouse F_28 RD1.svs	69_22 Mouse G_D28 RD1.svs	70_22 Mouse H_D28 RD1.svs	71_22 Mouse I_D28 RD1.svs	72_22 Mouse J_D28 RD1.svs
**Name**	PathAnnotationObject	PathAnnotationObject	PathAnnotationObject	PathAnnotationObject	PathAnnotationObject	PathAnnotationObject	PathAnnotationObject	PathAnnotationObject	PathAnnotationObject	PathAnnotationObject
**Class**	null	null	null	null	null	null	null	null	null	null
**Parent**	Image	Image	Image	Image	Image	Image	Image	Image	Image	Image
**ROI**	Geometry	Geometry	Geometry	Geometry	Geometry	Geometry	Geometry	Geometry	Geometry	Geometry
**Centroid X µm**	5274,5479	31983,5656	3455,9723	3202,8339	30399,7202	5047,5236	4358,6687	27376,6755	37887,3168	30239,575
**Centroid Y µm**	3708,2592	5352,1041	4747,007	3579,2731	4045,201	2663,6133	3464,1392	4968,8841	4872,8286	4738,7727
**Num Detections**	33180	28991	40611	35583	44229	27926	31526	30252	35173	42528
**Area µm^2**	9340376,944	7963355,428	11466637,76	9357174,045	11059952,38	8681839,675	8658602,369	9439663,017	9308038,14	13131755,95
**Perimeter µm**	39039,1877	25237,5119	19049,6505	31882,1086	32687,4775	29225,2462	29477,8907	19631,7072	25539,7005	26670,5019
**Image**	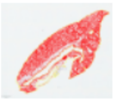	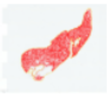	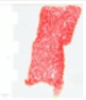	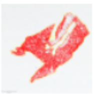	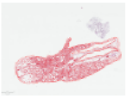	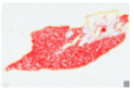	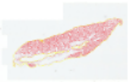	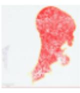	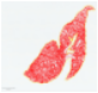	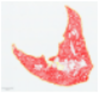
**Cells per um^2**	0,003552319	0,003640551	0,003541666	0,003802751	0,003999023	0,003216599	0,003641003	0,003204775	0,003778777	0,003238562
**% cells per um^2**	0,355231916	0,36405508	0,354166591	0,380275068	0,399902264	0,321659937	0,364100332	0,320477542	0,377877695	0,323856156
**Lesion detection**										
**Image**	63_22 Mouse A_D28 RD1.svs					68_22 Mouse F_28 RD1.svs				
**Name**	PathAnnotationObject					PathAnnotationObject				
**Class**	null					null				
**Parent**	Image					Image				
**ROI**	Polygon					Polygon				
**Centroid X µm**	5310,4247					6561,6432				
**Centroid Y µm**	4078,7091					1984,9738				
**Num Detections**	502					392				
**Area µm^2**	34523,5463	0	0	0	0	24851,2852	0	0	0	0
**Perimeter µm**	748,8736	0	0	0	0	689,8542	0	0	0	0
**Image**		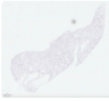			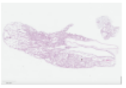					
**rea lung involvem**	0,3696162	0	0	0	0	0,286244461	0	0	0	0

Disease Models & Mechanisms • Supplementary information

H37RV-WT DAY42

134_22 Mouse D42 MB H37 Rv.svs	135_22 Mouse D42 MC H37 Rv.svs	136_22 Mouse D42 MD H37 Rv.svs	137_22 Mouse D42 ME H37 Rv.svs	138_22 Mouse D42 MF H37 Rv.svs	139_22 Mouse D42 MG H37 Rv.svs	140_22 Mouse D42 MH H37 Rv.svs	141_22 Mouse D42 MI H37 Rv .svs	142_22 Mouse D42 MJ H37 Rv .svs
PathAnnotationObject	PathAnnotationObject	PathAnnotationObject	PathAnnotationObject	PathAnnotationObject	PathAnnotationObject	PathAnnotationObject	PathAnnotationObject	PathAnnotationObject
null	null	null	null	null	null	null	null	null
Image	Image	Image	Image	Image	Image	Image	Image	Image
Geometry	Polygon	Polygon	Geometry	Geometry	Polygon	Geometry	Geometry	Geometry
29158,1264	4344,3901	4402,3993	5078,727	28852,7733	26933,3476	5724,8801	5736,6136	4866,4307
3223,2751	3869,2383	4272,63	1636,8938	4047,0214	6492,3661	2264,8446	7260,6789	3997,0319
102137	86351	66266	46934	59036	72939	44489	58750	68589
19138464,13	17565122,75	14773386,49	14180087,53	15760779,39	15550127,28	15344650,93	13840220,51	18049547,75
27035,2218	21144,7237	19843,2284	30289,4108	21365,5858	19826,9288	21267,1614	29348,4668	32530,5349
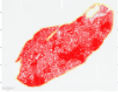	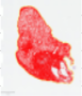	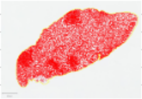	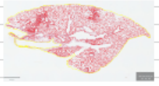	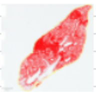	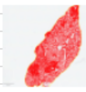	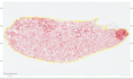	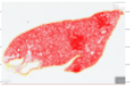	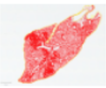
0,00533674	0,004916049	0,004485498	0,003309853	0,003745754	0,004690573	0,002899317	0,004244875	0,00380004
0,533673963	0,491604877	0,44854983	0,330985263	0,374575385	0,469057254	0,289931652	0,424487457	0,380003981
134_22 Mouse D42 MB H37 Rv.svs	135_22 Mouse D42 MC H37 Rv.svs	136_22 Mouse D42 MD H37 Rv.svs	137_22 Mouse D42 ME H37 Rv.svs	138_22 Mouse D42 MF H37 Rv.svs	139_22 Mouse D42 MG H37 Rv.svs	140_22 Mouse D42 MH H37 Rv.svs	141_22 Mouse D42 MI H37 Rv .svs	142_22 Mouse D42 MJ H37 Rv .svs
PathAnnotationObject	PathAnnotationObject	PathAnnotationObject	PathAnnotationObject	PathAnnotationObject	PathAnnotationObject	PathAnnotationObject	PathAnnotationObject	PathAnnotationObject
null	null	null	null	null	null	null	null	null
Image	Image	Image	Image	Image	Image	Image	Image	Image
Geometry	Geometry	Geometry	Geometry	Geometry	Geometry	Polygon	Geometry	Geometry
29156,2077	4645,2566	3259,2981	3786,3068	28882,9123	26578,9437	9526,589	6861,0533	3868,8642
3859,9564	4890,8803	4408,0714	1075,6824	3261,6458	6391,1455	1975,8752	7217,2228	4359,4128
34484	33651	15268	6123	19045	19935	3781	10013	21553
3521489,924	4396046,855	1591355,732	686530,018	2259103,457	2358964,438	393677,4482	1150892,214	2901942,95
38247,87	18394,1566	14754,7047	8250,0809	20930,3256	17613,0028	2984,6467	10578,9491	23811,2445
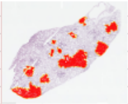	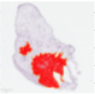	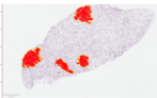	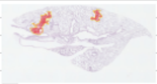	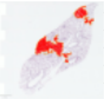	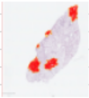		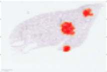	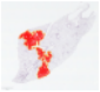
18,40006544	25,02713427	10,77177351	4,841507619	14,33370395	15,17006514	2,565567963	8,315562697	16,0776491

Disease Models & Mechanisms • Supplementary information

H37RV-RD1DAY42

Image	123_22 Mouse D42 MA RD1.svs	124_22 Mouse D42 MB RD1.svs	125_22 Mouse D42 MC RD1.svs	126_22 Mouse D42 MD RD1.svs	127_22 Mouse D42 ME RD1.svs	128_22 Mouse D42 MF RD1.svs	129_22 Mouse D42 MG RD1.svs	130_22 Mouse D42 MH RD1.svs	131_22 Mouse D42 MI RD1.svs	132_22 Mouse D42 MJ
Name	PathAnnotationObject	PathAnnotationObject	PathAnnotationObject	PathAnnotationObject	PathAnnotationObject	PathAnnotationObject	PathAnnotationObject	PathAnnotationObject	PathAnnotationObject	PathAnnotationOb
Class	null	null	null	null	null	null	null	null	null	null
Parent	Image	Image	Image	Image	Image	Image	Image	Image	Image	Image
ROI	Geometry	Geometry	Geometry	Geometry	Geometry	Geometry	Geometry	Geometry	Geometry	Geometry
Centroid X µm	5261,6464	31301,1208	30853,8267	6028,2672	3524,2913	27200,5834	29223,7338	6300,6375	4624,3032	4581,7873
Centroid Y µm	6296,0797	4903,2929	5814,3664	5155,0966	5049,1239	4296,645	7247,7542	4573,764	4475,0266	4528,8678
Num Detections	33160	47702	60916	32704	42691	39422	58573	54100	48221	43736
Area µm^2	7348660,668	13002245,75	11588093,06	9319919,722	12440124,63	12952116,66	15264159,88	12770627,6	13614104,54	15154034,78
Perimeter µm	26692,5948	26372,2978	22975,0146	19278,9151	36915,5485	31954,073	31086,7026	28800,7461	28810,2406	36123,3016
Image	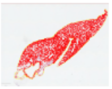	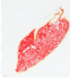	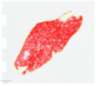	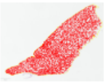	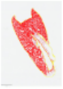	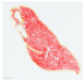	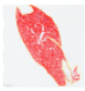	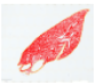	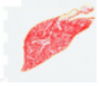	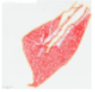
Cells per um^2	0,004512387	0,003668751	0,005256775	0,003509043	0,003431718	0,003043672	0,003837289	0,004236284	0,003541988	0,002886096
% cells per um^2	0,451238688	0,366875084	0,525677518	0,35090431	0,343171803	0,30436724	0,383728947	0,423628358	0,354198837	0,288609606
Lesion detection										
Image										
Name										
Class										
Parent										
ROI										
Centroid X µm										
Centroid Y µm										
Num Detections	0	0	0	0	0	0	0	0	0	0
Area µm^2	0	0	0	0	0	0	0	0	0	0
Perimeter µm										
Image	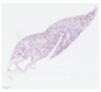	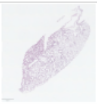	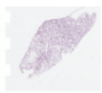				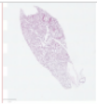	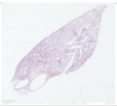	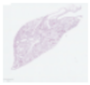	
% area lung involvement	0	0	0	0	0	0	0	0	0	0

H37RV-WT DAY 70

**Image**	153_22 Mouse D70 MA H37 Rv.svs	154_22 Mouse D70 MB H37 Rv.svs	155_22 Mouse D70 MC H37 Rv .svs	156_22 Mouse D70 MD H37 Rv.svs	157_22 Mouse D70 ME H37 Rv.svs	158_22 Mouse D70 MF H37 Rv.svs	159_22 Mouse D70 MG H37 Rv.svs	160_22 Mouse D70 MH H37 Rv .svs	161_22 Mouse D70 MI H37 Rv.svs	162_22 Mouse D70 [illegible]vs
**Name**	PathAnnotationObject	PathAnnotationObject	PathAnnotationObject	PathAnnotationObject	PathAnnotationObject	PathAnnotationObject	PathAnnotationObject	PathAnnotationObject	PathAnnotationObject	PathAnnotatio[illegible]
**Class**	null	null	null	null	null	null	null	null	null	null
**Parent**	Image	Image	Image	Image	Image	Image	Image	Image	Image	Image
**ROI**	Geometry	Geometry	Geometry	Geometry	Geometry	Geometry	Geometry	Geometry	Geometry	Geometr[illegible]
**Centroid X µm**	29595,269	5139,1572	6450,6301	4916,0894	4354,6916	5615,8881	4186,6236	4661,6026	4291,3751	3079,467[illegible]
**Centroid Y µm**	5476,3694	3703,4441	2515,2601	9573,1037	8746,3348	4711,6879	3910,9814	5384,0448	4949,0925	5988,781[illegible]
**Num Detections**	109397	104576	119523	83042	97400	131393	54838	91852	60260	65987
**Area µm^2**	23418274,96	18163201,93	20633328,56	16372730,85	20804487,86	19895985,91	14392137,16	16396458,56	14338423,6	15082681,[illegible]
**Perimeter µm**	36282,1409	27325,0005	27339,5333	30594,82	21925,5983	26834,3549	22337,7477	24512,0843	20874,056	25732,32[illegible]
**Image**	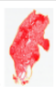	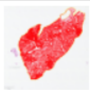	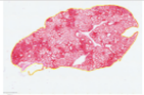	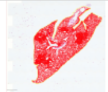	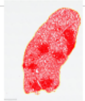	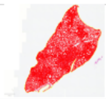	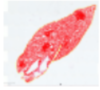	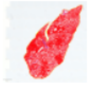	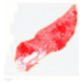	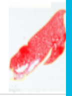
**Cells per um^2**	0,004671437	0,005757575	0,005792715	0,00507197	0,004681682	0,006603995	0,003810275	0,005601941	0,004202694	0,0043750[illegible]
**% cells per um^2**	0,467143716	0,575757515	0,57927154	0,507197002	0,468168218	0,660399543	0,381027497	0,56019414	0,420269352	0,4375017[illegible]
**Lesion detection**										
**Image**	153_22 Mouse D70 MA H37 Rv.svs	154_22 Mouse D70 MB H37 Rv.svs	155_22 Mouse D70 MC H37 Rv .svs	156_22 Mouse D70 MD H37 Rv.svs	157_22 Mouse D70 ME H37 Rv.svs	158_22 Mouse D70 MF H37 Rv.svs	159_22 Mouse D70 MG H37 Rv.svs	160_22 Mouse D70 MH H37 Rv .svs	161_22 Mouse D70 MI H37 Rv.svs	162_22 Mouse D70 [illegible]vs
**Name**	Positive	PathAnnotationObject	PathAnnotationObject	PathAnnotationObject	PathAnnotationObject	PathAnnotationObject	PathAnnotationObject	PathAnnotationObject	PathAnnotationObject	PathAnnotatio[illegible]
**Class**	Positive	null	null	null	null	null	null	null	null	null
**Parent**	Image	Image	Image	Image	Image	Image	Image	Image	Image	Image
**ROI**	Geometry	Geometry	Geometry	Geometry	Geometry	Geometry	Geometry	Geometry	Geometry	Geometr[illegible]
**Centroid X µm**	29815,4923	4689,5343	5961,2658	4401,7401	4439,3356	5882,362	4297,4516	4308,8856	4361,4061	3592,725[illegible]
**Centroid Y µm**	4801,3128	3804,0234	2288,8101	9696,9389	9577,6634	4747,0008	3453,4055	5855,0195	4894,5059	6014,116[illegible]
**Num Detections**	37449	33850	42897	24990	37924	57465	10858	52054	22068	16746
**Area µm^2**	4668755,085	3366401,79	4798643,305	2344344,728	4225759,948	6174826,355	1195625,41	2778889,453	2497449,825	1917224,7[illegible]
**Perimeter µm**	35175,5414	39555,3814	47966,0079	18753,2213	24826,9917	39690,4434	12756,5686	29635,7385	20924,0073	13499,44[illegible]
**Image**	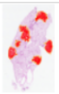	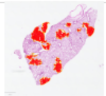	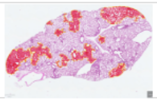	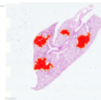	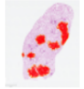	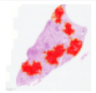	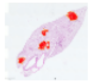	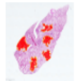	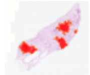	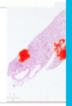
**% area lung involvement**	19,93637487	18,53418689	23,25675807	14,31859321	20,31177108	31,03553845	8,30749038	16,94810768	17,41788285	12,71143[illegible]

Disease Models & Mechanisms • Supplementary information

H37RV-RD1DAY70

Image	143_22 Mouse D70 MA RD1.svs	144_22 Mouse D70 MB RD1.svs	145_22 Mouse D70 MC RD1.svs	146_22 Mouse D70 MD RD1.svs	147_22 Mouse D70 ME RD1.svs	148_22 Mouse D70 MF RD1.svs	149_22 Mouse D70 MG RD1.svs	150_22 Mouse D70 MH RD1.svs	151_22 Mouse D70 MI RD1.svs	152_22 Mouse D70 MJ RD1.svs
Name	PathAnnotationObject	PathAnnotationObject	PathAnnotationObject	PathAnnotationObject	PathAnnotationObject	PathAnnotationObject	PathAnnotationObject	PathAnnotationObject	PathAnnotationObject	PathAnnotationObject
Class	null	null	null	null	null	null	null	null	null	null
Parent	Image	Image	Image	Image	Image	Image	Image	Image	Image	Image
ROI	Geometry	Geometry	Geometry	Geometry	Geometry	Geometry	Geometry	Geometry	Geometry	Geometry
Centroid X µm	4672,0151	31759,0167	31164,2521	34890,0416	34421,6174	30458,3821	32671,9456	31596,0409	4306,8325	30325,9219
Centroid Y µm	4645,3252	3965,5344	6237,9899	4752,3667	6534,8051	2606,2262	8515,4834	3774,8845	5534,4052	5329,4426
Num Detections	49246	49264	53499	46395	57761	50810	47592	25127	42555	15818
Area µm^2	12697828,42	13283400,84	16347438,19	21447720,41	15941313,03	15568915,53	13166584,12	9296668,275	14074545,91	12463210,62
Perimeter µm	29505,2842	31678,7354	27364,9455	33103,7711	52750,3425	32761,7901	29192,6373	32898,6412	32646,6752	40581,2705
Image	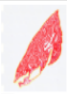	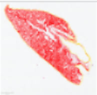	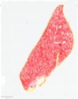	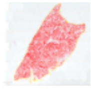	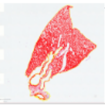	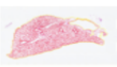	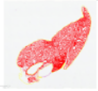	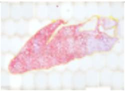	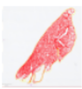	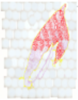
Cells per um^2	0,003878301	0,003708689	0,003272623	0,002163167	0,003623353	0,003263554	0,003614605	0,002702796	0,003023543	0,001269175
% cells per um^2	0,387830095	0,37086888	0,327262286	0,216316695	0,362335273	0,326355422	0,361460494	0,270279623	0,302354337	0,126917537
Lesion detection										
Image							149_22 Mouse D70 MG RD1.svs			
Name							PathAnnotationObject			
Class							null			
Parent							Image			
ROI							Polygon			
Centroid X µm							32912,5577			
Centroid Y µm							9559,5728			
Num Detections	0	0	0	0	0	0	7589	0	0	0
Area µm^2	0	0	0	0	0	0	728919,6758	0	0	0
Perimeter µm							4774,9535			
Image	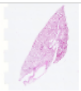	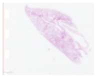			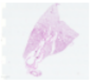	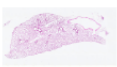	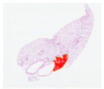	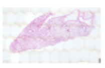	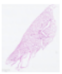	
% area lung involvement	0	0	0	0	0	0	5,536133513	0	0	0

Disease Models & Mechanisms • Supplementary information

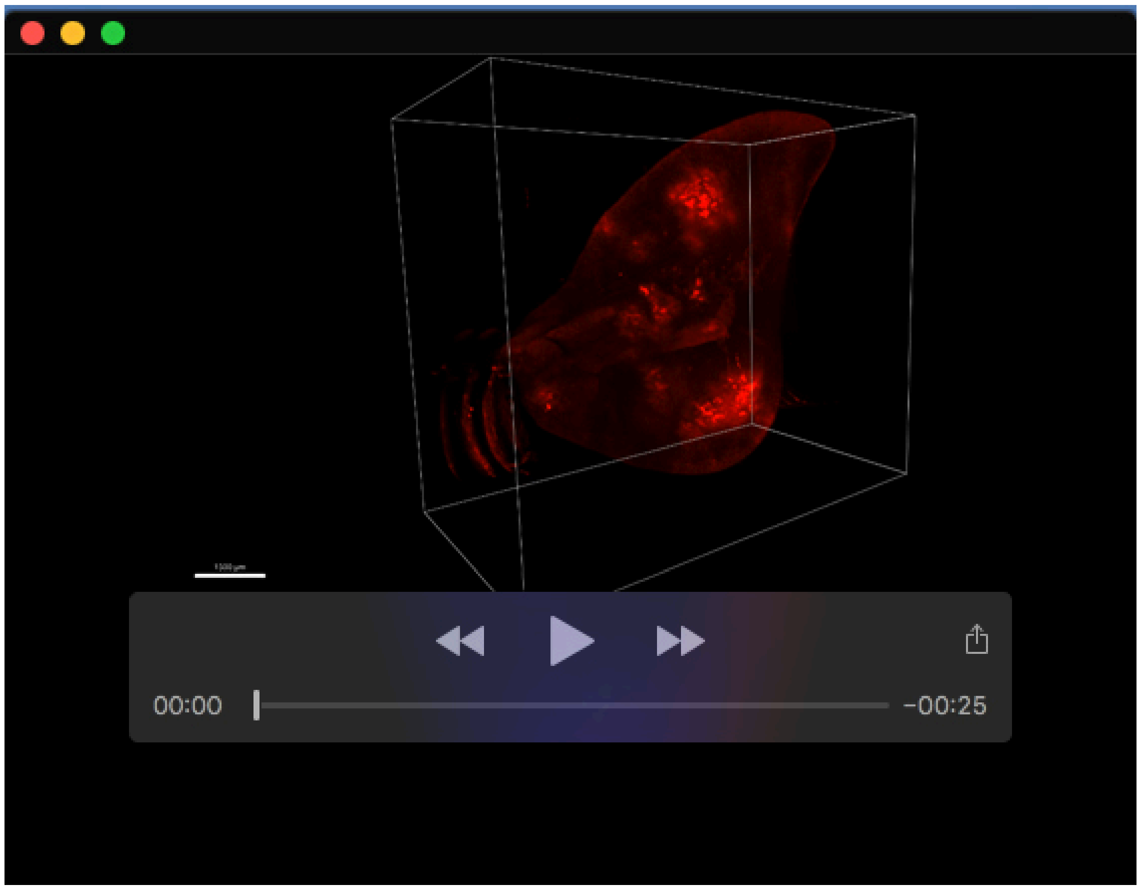

Movie 1. 3D projection of cleared lung lobe infected with E2Crimson *Mtb* WT (42 days post-infection) imaged using a LaVision Ultramicroscope II Light Sheet Microscope (Miltenyi Biotec) using low magnification (0.63 X zoom) and static light sheet.

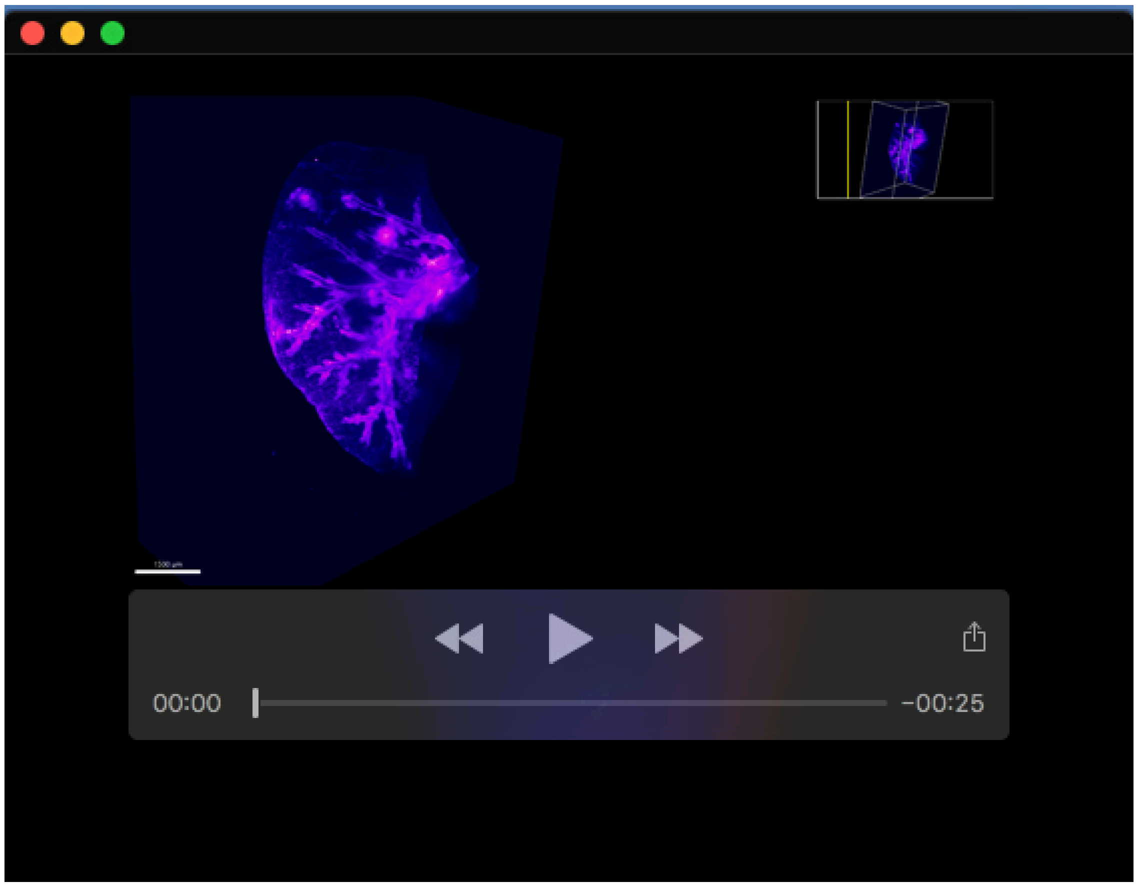

Movie 2. 3D projection of E2Crimson *Mtb* WT H37Rv-infected C3HeB/FeJ mice at 28 days post-infection, with small foci of CD11b positive cells stemming from an upper branch.

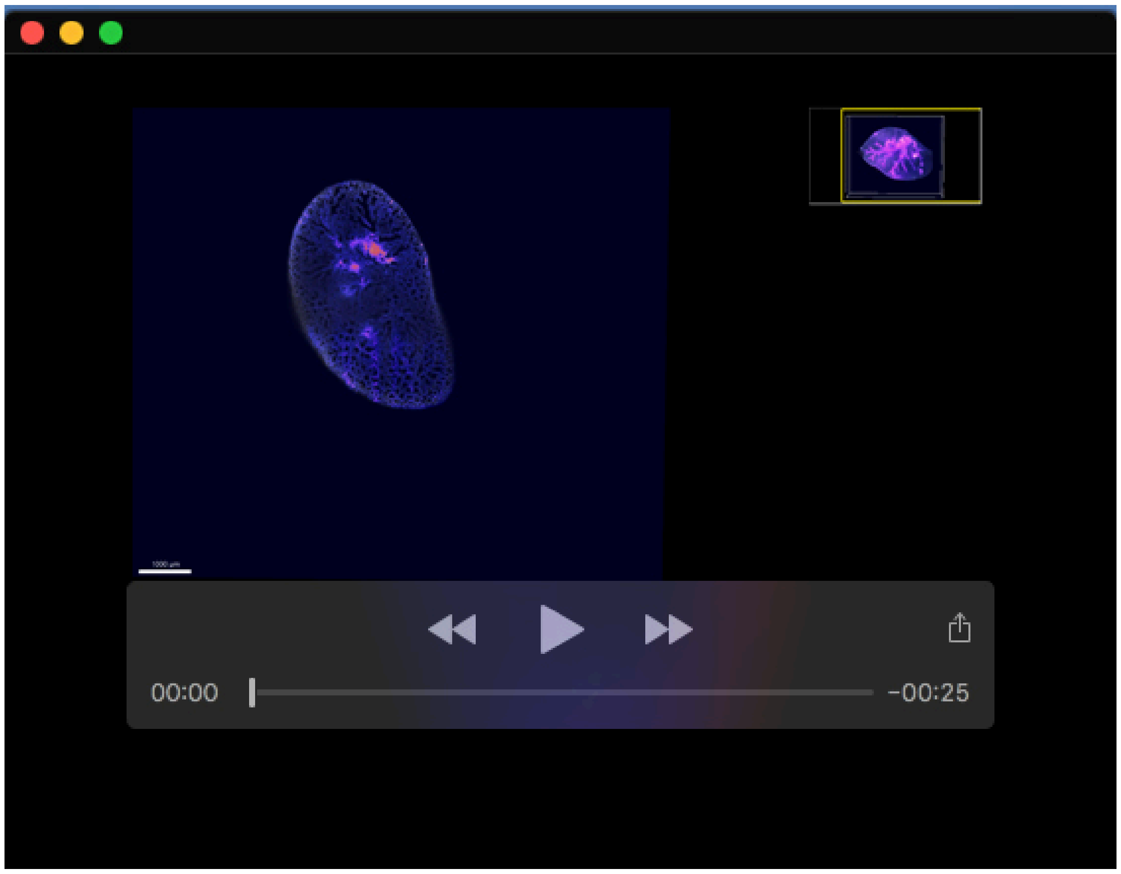

Movie 3. Orthogonal projection stack of E2Crimson *Mtb* WT H37Rv-infected C3HeB/FeJ mice at 28 days post-infection.

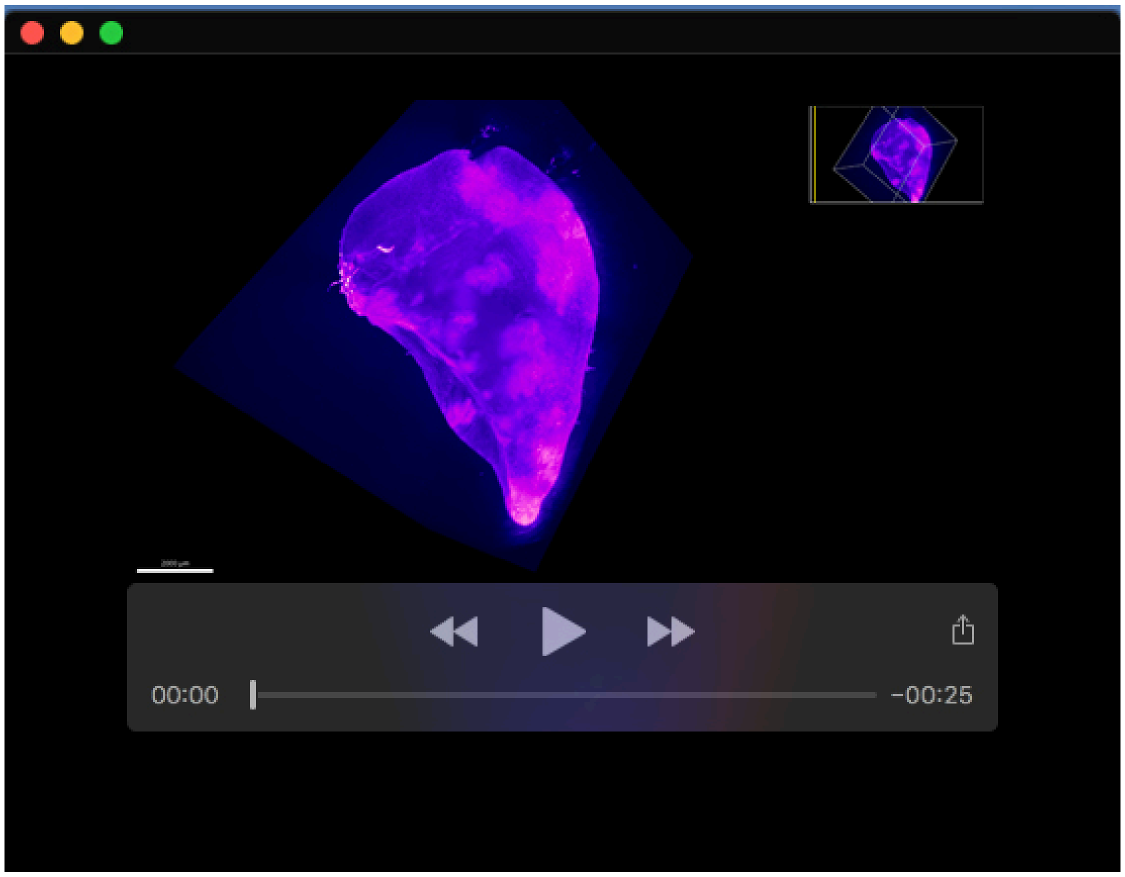

Movie 4. 3D projection of E2Crimson *Mtb* WT H37Rv-infected C3HeB/FeJ mice at 42 days post-infection.

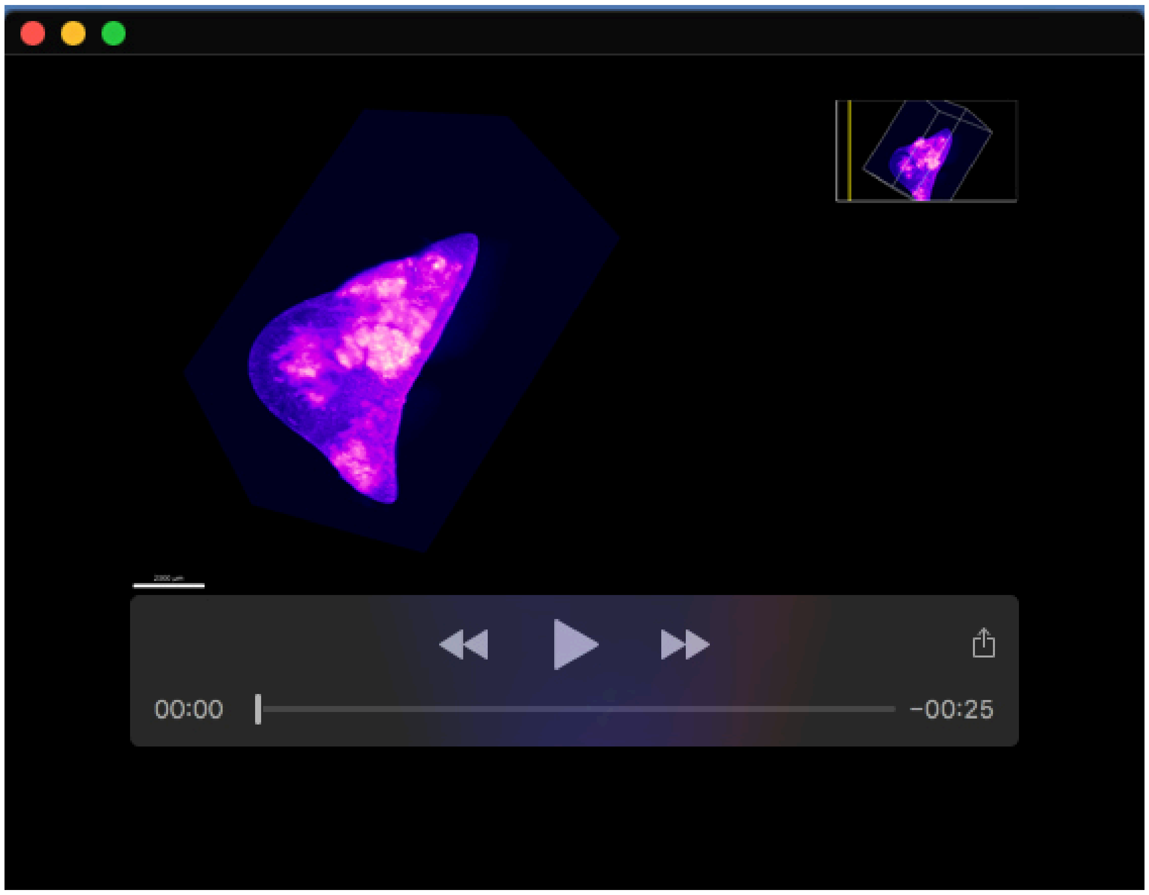

Movie 5. 3D projection of E2Crimson *Mtb* WT H37Rv-infected C3HeB/FeJ mice at 70 days post-infection.

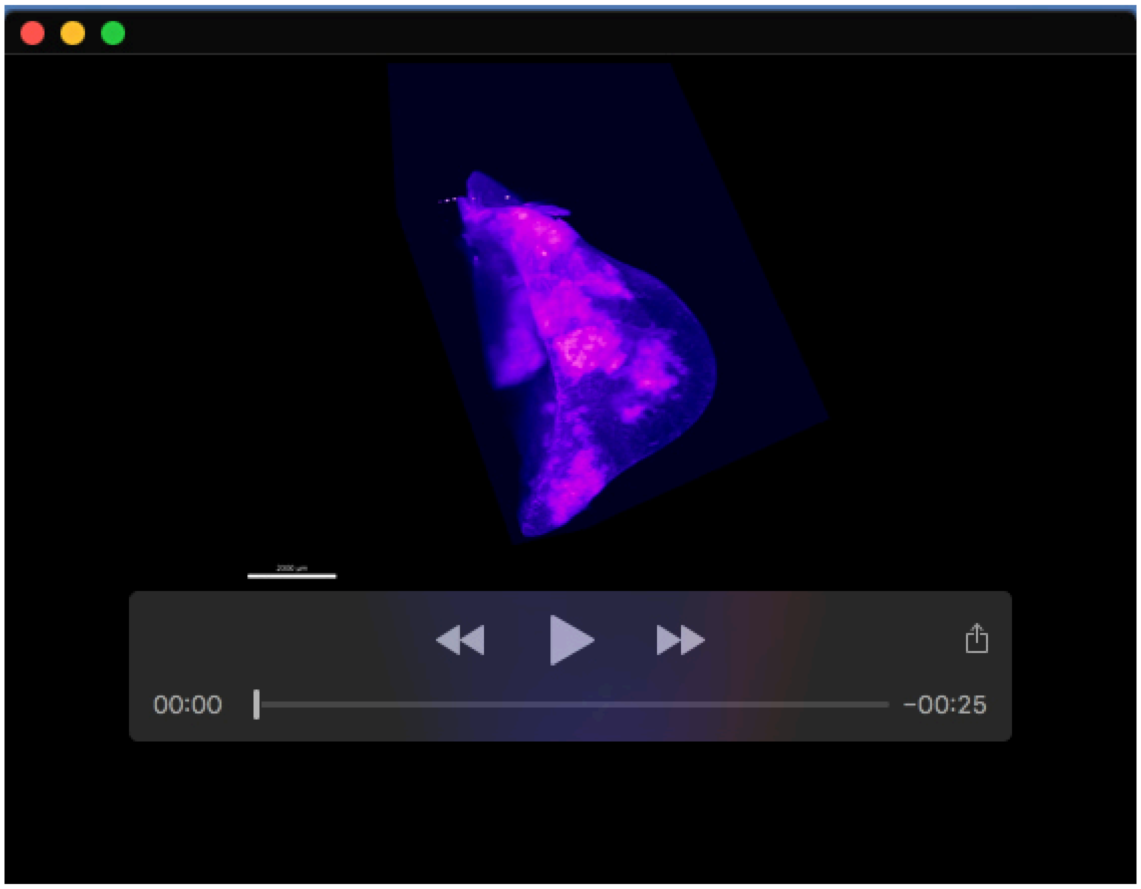

Movie 6. 3D render of lesion volume and architecture in E2Crimson *Mtb* WT H37Rv-infected C3HeB/FeJ mice at 70 days post-infection.

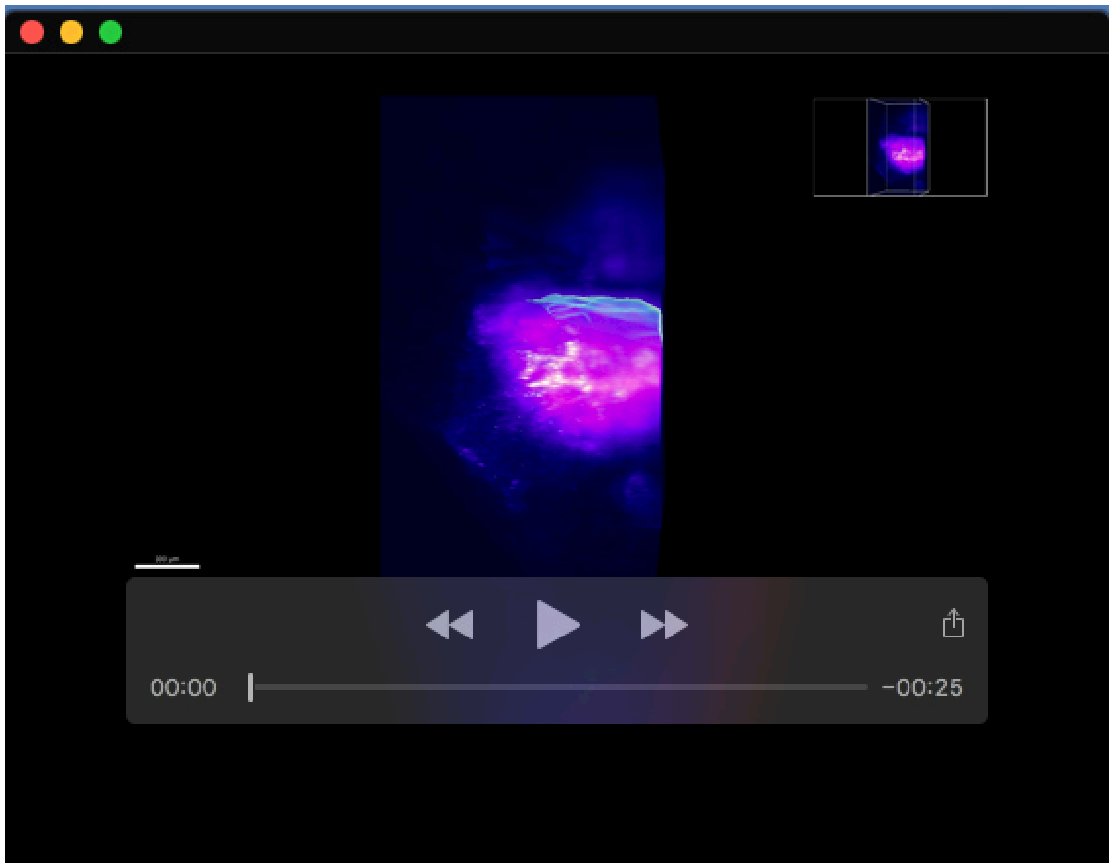

Movie 7. 3D render of individual lesion volume and infecting *Mtb* cells in E2Crimson *Mtb* H37Rv-infected C3HeB/FeJ mice at 70vdays post-infection.

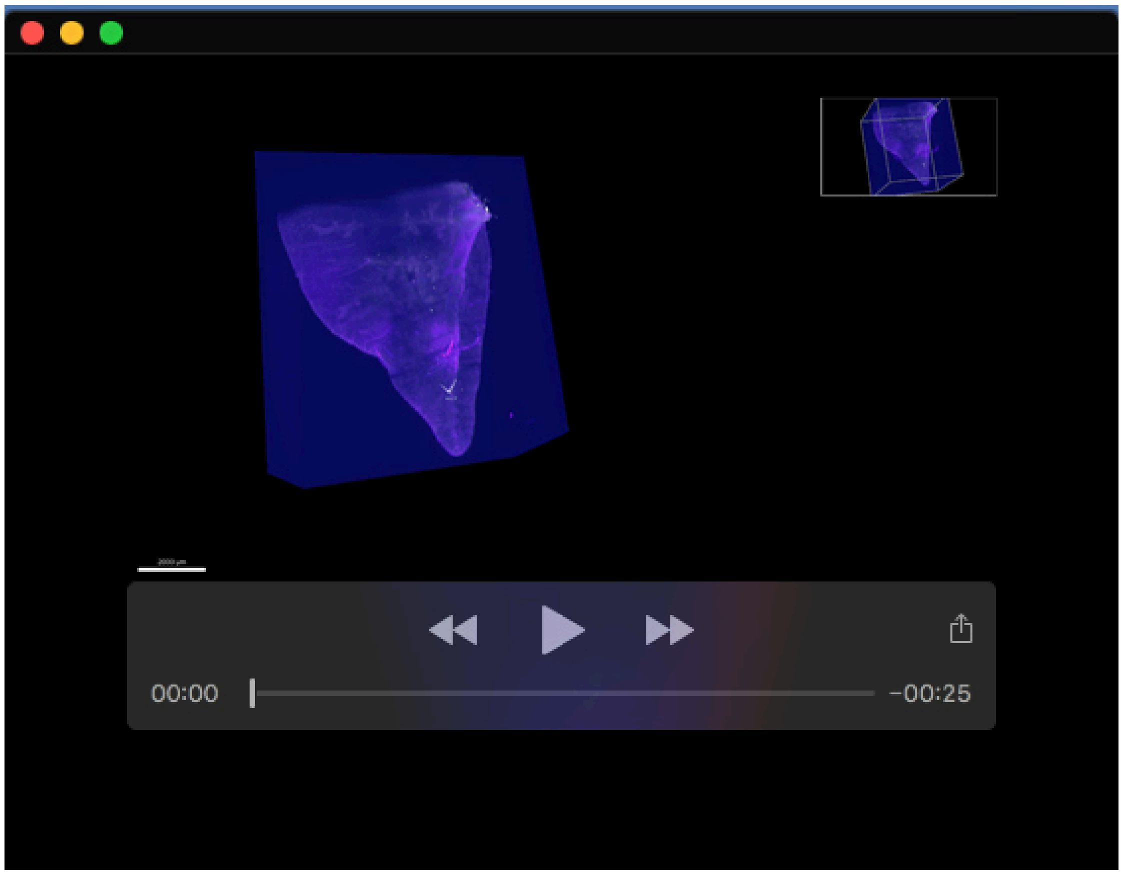

Movie 8. 3D projection of E2Crimson *Mtb* ΔRD1 H37Rv-infected C3HeB/FeJ mice at 28 days post-infection.

Disease Models & Mechanisms • Supplementary information

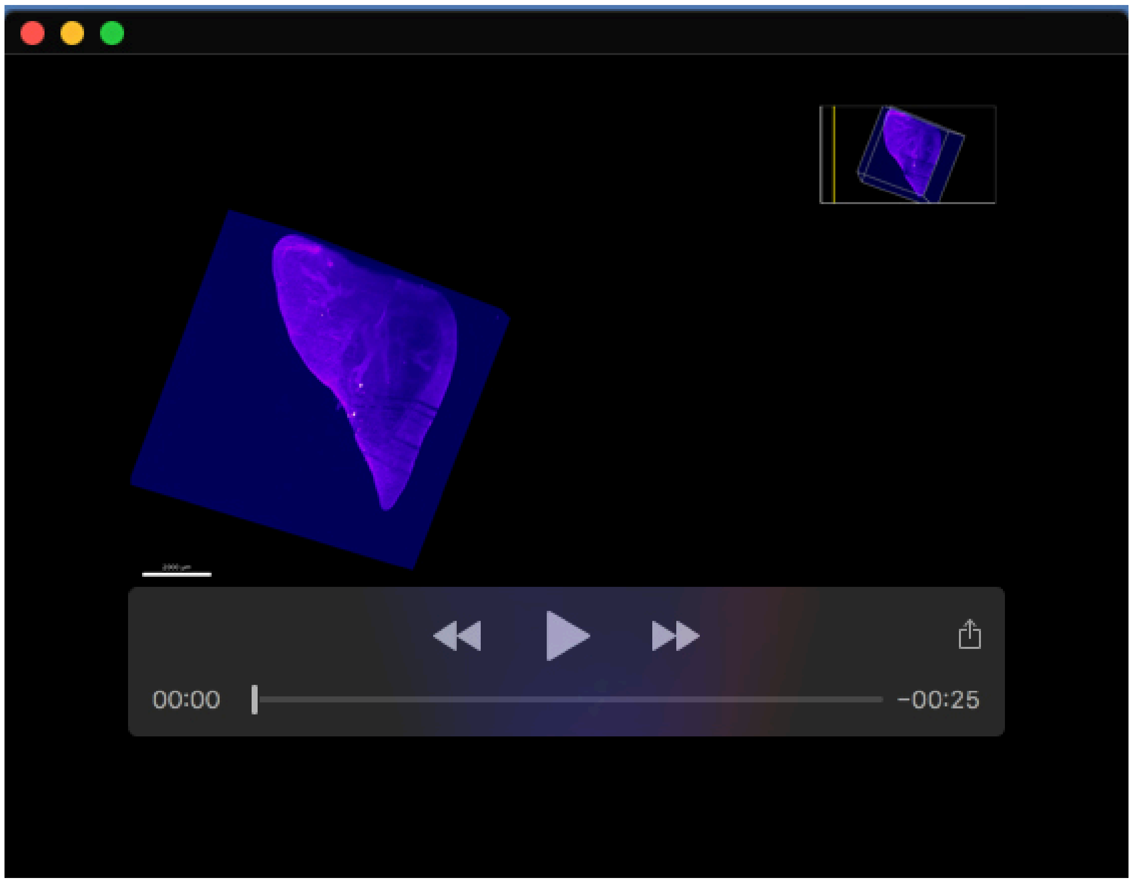

Movie 9. 3D projection of E2Crimson *Mtb* ΔRD1 H37Rv-infected C3HeB/FeJ mice at 42 days post-infection.

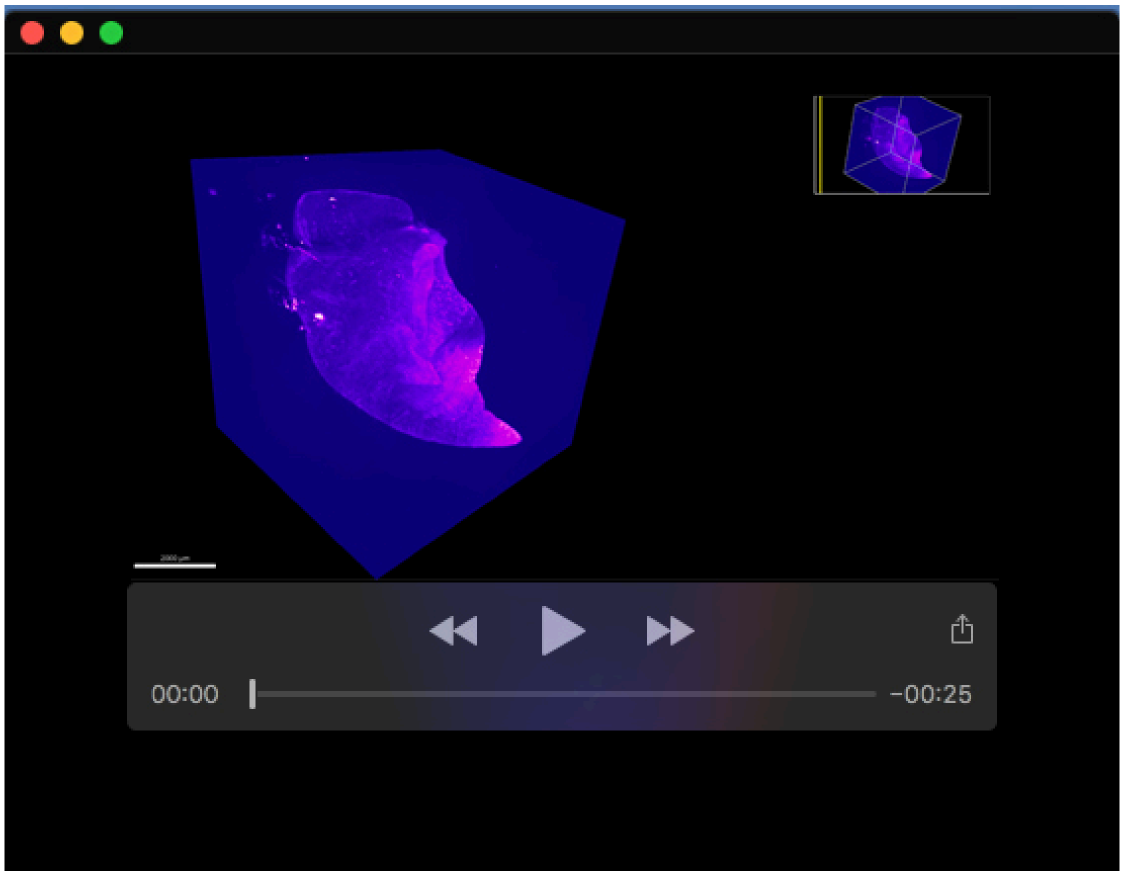

Movie 10. 3D projection of E2Crimson *Mtb* ΔRD1 H37Rv-infected C3HeB/FeJ mice at 70 days post-infection.
